# Therapeutic effects of platelet-derived extracellular vesicles on viral myocarditis correlate with biomolecular content

**DOI:** 10.3389/fimmu.2024.1468969

**Published:** 2025-01-06

**Authors:** Danielle J. Beetler, Presley Giresi, Damian N. Di Florio, Jessica J. Fliess, Elizabeth J. McCabe, Molly M. Watkins, Vivian Xu, Matthew E. Auda, Katelyn A. Bruno, Emily R. Whelan, Stephen P. C. Kocsis, Brandy H. Edenfield, Sierra A. Walker, Logan P. Macomb, Kevin C. Keegan, Angita Jain, Andrea C. Morales-Lara, Isha Chekuri, Anneliese R. Hill, Houssam Farres, Joy Wolfram, Atta Behfar, Paul G. Stalboerger, Andre Terzic, Leslie T. Cooper, DeLisa Fairweather

**Affiliations:** ^1^ Department of Cardiovascular Medicine, Mayo Clinic, Jacksonville, FL, United States; ^2^ Center for Clinical and Translational Science, Mayo Clinic, Rochester, MN, United States; ^3^ Mayo Clinic Graduate School of Biomedical Sciences, Mayo Clinic, Rochester, MN, United States; ^4^ Division of Cardiovascular Medicine, University of Florida, Gainesville, FL, United States; ^5^ Department of Cancer Biology, Mayo Clinic, Jacksonville, FL, United States; ^6^ Center for Systems Biology, Massachusetts General Hospital, Boston, MA, United States; ^7^ Department of Molecular Pharmacology & Experimental Therapeutics, Mayo Clinic, Rochester, MN, United States; ^8^ Department of Vascular Surgery, Mayo Clinic, Jacksonville, FL, United States; ^9^ School of Chemical Engineering, The University of Queensland, Brisbane, QLD, Australia; ^10^ Australian Institute for Bioengineering and Nanotechnology, The University of Queensland, Brisbane, QLD, Australia; ^11^ Van Cleve Cardiac Regenerative Medicine Program, Mayo Clinic Center for Regenerative Medicine, Rochester, MN, United States; ^12^ Department of Clinical Genomics, Mayo Clinic, Rochester, MN, United States; ^13^ Department of Immunology, Mayo Clinic, Jacksonville, FL, United States

**Keywords:** coxsackievirus B3, innate immunity, complement, TLR4, sex differences, microRNA

## Abstract

**Introduction:**

Extracellular vesicles (EVs) can potently inhibit inflammation yet there is a lack of understanding about the impact of donor characteristics on the efficacy of EVs. The goal of this study was to determine whether the sex and age of donor platelet-derived EVs (PEV) affected their ability to inhibit viral myocarditis.

**Methods:**

PEV, isolated from men and women of all ages, was compared to PEV obtained from women under 50 years of age, which we termed premenopausal PEV (pmPEV). Because of the protective effect of estrogen against myocardial inflammation, we hypothesized that pmPEV would be more effective than PEV at inhibiting myocarditis. We injected PEV, pmPEV, or vehicle control in a mouse model of viral myocarditis and examined histology, gene expression, protein profiles, and performed proteome and microRNA (miR) sequencing of EVs.

**Results:**

We found that both PEV and pmPEV significantly inhibited myocarditis; however, PEV was more effective, which was confirmed by a greater reduction of inflammatory cells and proinflammatory and profibrotic markers determined using gene expression and immunohistochemistry. Proteome and miR sequencing of EVs revealed that PEV miRs specifically targeted antiviral, Toll-like receptor (TLR)4, and inflammasome pathways known to contribute to myocarditis while pmPEV contained general immunoregulatory miRs.

**Discussion:**

These differences in EV content corresponded to the differing anti-inflammatory effects of the two types of EVs on viral myocarditis.

## 1 Introduction

Extracellular vesicles (EVs) are biological nanoparticles that can be used therapeutically because of their ability to act as biocompatible disease modulators. EVs function as important systems for cellular communication in the body, encapsulating autocrine, paracrine, and endocrine messages that regulate local and systemic responses (1, 2). The umbrella term ‘EV’ refers to lipid bilayer encapsulated, functional nanoparticles from two main biogenesis categories: either an invagination of the plasma membrane and intracellular processing into vesicles (exosomes), or an outward budding of the plasma membrane during specific cell responses, including apoptosis (ectosomes or microvesicles) (3, 4). However, these categories are distinct in name and biogenesis only, as functional differences cannot yet be ascertained (4). There are currently no accepted exosome or microvesicle-specific markers, and thus EVs are characterized as bulk, heterogenous products (3, 5). The notorious heterogeneity of EVs has created significant limitations to their use as therapies. EVs contain an unlimited variety of functional contents including DNA, mRNA, microRNA (miRs), glycans, and proteins (5, 6). Depending on the state of the parent cell at the time the EV is formed, they may also contain cellular organelles or portions of organelles like mitochondria (3, 7). EV heterogeneity is further induced by differing isolation procedures, storage temperatures, and processing times, all of which can alter the composition of EVs and complicate batch-to-batch consistency of therapeutic products (3, 5).

Another understudied source of heterogeneity is donor origin. The importance of identifying an ‘ideal donor’ is not a new topic, as the regenerative medicine field has documented concerns regarding this issue for many years (8, 9). However, poor understanding regarding the impact of donor selection on EV characteristics remains a significant gap in knowledge. The body of literature surrounding preclinical EV therapies is growing; however, donor selection criteria other than age is a largely unexplored topic (10, 11). Regardless of whether EVs are derived from cell culture or extracted directly from patient fluids or tissues, identification of ideal donors or ideal donor subsets that confer maximum therapeutic potency is essential.

We recently reported that platelet-derived EVs were able to reduce myocardial inflammation and fibrosis and improve cardiac function in a translational model of viral myocarditis (12). Myocarditis is defined as inflammation of the heart and is commonly associated with viral infections such as SARS-CoV-2 and coxsackie B virus (CVB) infections (13–15). Our group uses an autoimmune CVB3 model of myocarditis, where a mild viral infection with a human derived-CVB3 strain acts as an adjuvant to induce myocarditis (16). The model is highly translational, matching the time-course, clinical features, pathogenesis of disease, and biomarkers of human lymphocytic myocarditis, including progression to dilated cardiomyopathy (DCM) and heart failure in susceptible strains of mice (14, 17–19). Clinical myocarditis has been reported to have a sex ratio of around 3.5:1 male to female (14, 19). Our animal model of CVB3 myocarditis also displays a clear sex difference where the immune response by sex has been well characterized [reviewed in (13, 14)]. We, and others, have shown previously that testosterone increases CVB3 myocarditis while estrogen/17β1-estradiol is cardioprotective (14, 19–21). Key pathways that increase CVB3 myocarditis in males include Toll-like receptor (TLR)4 signaling on cardiac mast cells and macrophages, and inflammasome activation, which lead to elevated interleukin (IL)-1β levels that increase inflammation and remodeling/fibrosis, resulting in acute myocarditis and progression to DCM in white background mice (i.e., BALB/c, A/J) (14, 22, 23). Because of the strongly protective role of estrogen in myocarditis, here we investigated whether specific donor demographics (sex and age) would alter the ability of EVs to reduce myocarditis. We tested whether platelets obtained from women under the age of 50 would be more effective at reducing myocarditis than EVs obtained from the platelets of men and women of all ages.

## 2 Materials and methods

### 2.1 Ethics statement

Mice were maintained under pathogen-free conditions in the animal facility at Mayo Clinic Florida, with approval from the Institutional Animal Care and Use Committee (IACUC) at Mayo Clinic Florida for all procedures (IACUC A00002398). Mice were used and humanely euthanized according to the standards of the Guide for the Care and Use of Laboratory Animals of the National Institutes of Health and in accordance with practices of the Mayo Clinic Florida IACUC.

### 2.2 Platelet-derived EV products

Human EVs were obtained from pooled apheresis platelets from two groups of United States donors to make two analogous, but separate products. A batch of platelet-derived EVs (PEV) was created from 10 male and female donors of all ages, as previously described (24, 25). Similarly, a batch of EVs was created from 10 women under the age of 50 years, which we termed premenopausal PEV (pmPEV). Both PEV and pmPEV were processed in the same manner. Briefly, a clinical grade manufacturing protocol was used to pool conditioned medium from apheresis platelets and isolate exosomes according to US patent 20160324A1 (24, 25). Serial filtration and lyophilization processes yielded a bioactive extracellular vesicle cake in sterile vials, which was confirmed to be pyrogen free, without preservatives, and room temperature stable up to 24 months. The presence of platelet-derived EVs was confirmed using EV markers CD63 and CD9, and platelet marker CD41, as previously (24, 25). PEV is manufactured by Rion, Inc. (Rochester, Minnesota, USA) and is approved by the US Food and Drug Administration (FDA) for use as an investigational new drug in human clinical trials for wound healing (NCT04664738) and has received FDA approval for Phase 1 clinical trials in ischemic heart disease. pmPEV was produced specifically for use in this project. PEV and pmPEV were reconstituted in 1x sterile phosphate buffered saline (PBS) at 11.2 mg dry weight for PEV/mL and 8.0 mg dry weight for pmPEV/mL to achieve a final concentration of 1x10 ^12^ particles/mL for both products (24, 25).

### 2.3 Extracellular vesicle characterization

#### 2.3.1 Nanoparticle tracking analysis

Nanoparticle tracking analysis (NTA) was used to determine the size distribution and particle concentration of reconstituted PEV and pmPEV using a Nanosight NS300 V3.3.4 (Malvern Panalytical, Malvern, United Kingdom) for three replicates with measurement time at 60 s, flow rate 40 µL/min, camera level 11, and detection threshold 4, as previously (12).

#### 2.3.2 Tunable resistive pulse sensing

Tunable resistive pulse sensing (TRPS) was used as an orthogonal method for size and concentration measures and to analyze zeta potential using an Exoid (catalog# EX1, Izon Science, Boston, Massachusetts, USA) and a 200 nm nanopore (catalog#NP200, Izon Science, Boston, Massachusetts, USA). Briefly, samples were run through a nanopore which detects resistive pulses caused by single nanoparticles passing through the charged and pressurized system. Calibration using beads (catalog#CPC200, Izon Science, Boston, Massachusetts, USA) was performed prior to sample data acquisition according to manufacturer specification and software prompts. Samples were diluted in 1xPBS with the following measurement specifications and quality control reporting: PEV (stretch: 45.49 mm, pressure: 700 Pa, voltage: 700 mV, bandwidth filter: not applied, particle rate: 474.6 particles/min, average current: 140.08 nA, and average RMS noise: 16.82 pA), pmPEV (stretch: 45.49, pressure: 1000, voltage: 600, bandwidth filter: not applied, particle rate: 212.0, average current: 126.01, and average RMS noise: 19.25). Analysis of calibration and sample data was performed using Izon data suite software version 1.0.2.32.

#### 2.3.3 EV multiplex panel

The ProcartaPlex Human Exosome Characterization Panel 6-PlexEV multiplex assay (catalog#EPXX060-15845-901, Invitrogen, Waltham, Massachusetts, USA) was used to determine six markers: EV tetraspanins CD81, CD63, and CD9; cytosolic markers syntenin-1 and cytochrome c; and immune receptor very late antigen (VLA)-4. To make replicable comparisons, protein amounts of these markers in reconstituted PEV and pmPEV were calculated relative to amounts in a standardized EV reference control from the HCT116 human colorectal cell line (E1, catalog# 00000861, Oxford Nanopore, Oxford, Oxfordshire, United Kingdom). Plate measurements were made using a FLEXMAP 3D Luminex 200 (catalog#FLEXMAP-3D-RUO, R&D Systems, Minneapolis, Minnesota, USA) analyzed on The ProcartaPlex Analysis App on the ThermoFisher Connect cloud-based platform: https://apps.thermofisher.com/apps/procartaplex.

#### 2.3.4 Enzyme-linked immunosorbent assay

Enzyme-linked immunosorbent assays (ELISAs) were used to determine protein levels of lipoproteins apolipoprotein (Apo)A1 (catalog#DAPA10, R&D Systems, Minneapolis, Minnesota, USA) and ApoB (catalog#DAPB00, R&D Systems, Minneapolis, Minnesota, USA) in reconstituted PEV, pmPEV, and standardized control (E1, catalog# 00000861, Oxford Nanopore, Oxford, Oxfordshire, United Kingdom) samples, following kit specifications. Final protein concentrations were read at 450 nm by an 800 TS Microplate Reader (cat # 800TS-SN, BioTeK, Winooski, Vermont, USA). Protein concentrations were normalized to total amount of protein per sample in duplicate, calculated from a Bradford assay.

#### 2.3.5 Size-exclusion chromatography

Size-exclusion chromatography (SEC) was performed on PEV and pmPEV prior to visualization techniques (microscopy imaging) during characterization using an Automated Fraction Collector V1 (Izon Science, Boston, Massachusetts, USA) with a qEVoriginal column (70nm Legacy Column, cat# SP1, Izon Science, Boston, Massachusetts, USA). Briefly, following the machine default prompts for this column, 0.5 mL of reconstituted PEV or pmPEV were loaded to the top of the column and eluted into thirteen 0.5 mL fractions using a cryoprotective 5% sucrose buffer (26). Fractions 7-11, which contained enriched EVs were pooled and used for microscopy.

#### 2.3.6 Transmission electron microscopy

Transmission electron microscopy (TEM) imaging was conducted at the Mayo Clinic Microscopy and Cell Analysis Core (Rochester, Minnesota, USA) to visualize reconstituted PEV and pmPEV. Briefly, 3 µL of each reconstituted product was placed on a carbon-coated polymer film, mounted on a 200-mesh copper TEM grid, and allowed to thin and dry. 3 µL of negative stain (1% phosphotungstic acid) was added to the sample on the grid and allowed to thin and dry. Grids were loaded into a JEOL 1400 (JEOL USA, Inc., Peabody, Massachusetts, USA) transmission electron microscope operating at 80 KeV. Representative images were captured using a Gatan 832 Orius digital camera (Gatan, Pleasanton, California, USA).

#### 2.3.7 Western blot

After reconstitution, PEV and pmPEV were lysed in 1x radioimmunoprecipitation assay buffer (RIPA; catalog#89900, Pierce, Thermo-Scientific, Waltham, Massachusetts, USA) and 100X Halt™ Protease Inhibitor Cocktail (catalog#1862495, Pierce, Thermo-Scientific, Waltham, Massachusetts, USA), per manufacturer instructions. A bicinchoninic acid assay (BCA) (Pierce BCA Reagents A (catalog#23228) and B (catalog#1859078), Thermo-Scientific, Waltham, Massachusetts, USA) was used to determine lysate concentration. A total of 10.3 µg protein was loaded per lane in NuPAGE™ 4-12%, Bis-Tris, 1.0–1.5 mm, Mini Protein Gels (catalog# NP0322BOX, Thermo-Scientific, Waltham, Massachusetts, USA) and run at 165 volts in 1X NuPAGE™ 4-morpholinepropanesulfonic acid (MOPS) sodium dodecyl sulfate (SDS) Running Buffer (catalog#NP000102, Thermo-Scientific, Waltham, Massachusetts, USA) for 45 minutes. A Trans-Blot Turbo Transfer System was used to transfer protein to Nitrocellulose membranes using the with Mini 0.2 µm Nitrocellulose Transfer Packs (catalog#1704158, BioRad, Hercules, California, USA). Blots were blocked in 5% bovine serum albumin (BSA) in Tris-buffered saline with Tween 20 (TBST) before incubation in primary antibody solution (**Supplementary Table 1**). The blot was then incubated in secondary antibody solution Peroxidase AffiniPure Goat Anti-Mouse IgG (H+L) (catalog#115-035-003, 1:7500) or Peroxidase AffiniPure Goat Anti-Rabbit IgG (H+L) (catalog#111-035-003, 1:7500) (Jackson ImmunoResearch, West Grove, Pennsylvania, USA) in 10 mL of 5% milk in TBST and imaged using a XOMAT film imager (Carestream Health, Rochester, New York, USA).

#### 2.3.8 Tri-color direct stochastic optical reconstruction microscopy

The ONI Profiler Kit for tetraspanin profiling of EVs (catalog# 00000861, Oxford Nanopore, Oxford, Oxfordshire, United Kingdom) was used to visualize the provided standard control and SEC fractions 7-11 of PEV and pmPEV. Staining for tetraspanins CD9, CD63, and CD81 was performed according to manufacturer instructions and direct stochastic optical reconstruction microscopy (dSTORM) images of all samples were acquired on an Oxford NanoImager (ONI) Microscope (Oxford, Oxfordshire, United Kingdom) in total internal reflection fluorescence (TIRF) microscopy mode to provide a resolution of 20-30 nm. Images were acquired using the 488 and 647 lasers and laser power was increased until visible photoswitching of fluorophores was apparent while samples were covered in buffer (GLOX beta-mercapto-ethanol with Pyranose Oxidase as previously (27). *A* total of 4,000 images were acquired for each channel, and localizations were determined in CODI (Oxford Nanoimaging/ONI), with localizations filtered by frame index and p value, as previously described (12). Subsequent positivity analyses were performed on all samples to determine tetraspanin profiles.

### 2.4 *In vivo* treatments

Mice were obtained from the Jackson Laboratory (8-week-old adult male BALB/cJ strain #000651, Bar Harbor, Maine, USA) and were maintained under pathogen-free conditions in the Mayo Clinic Florida animal facility. Ten mice per group (control, PEV, pmPEV) were infected intraperitoneally (ip) with 10^3^ plaque forming units (PFU) of heart-passaged stock of coxsackievirus B3 (CVB3) on day 0, and myocarditis examined at day 10 post infection (pi). CVB3 (Nancy strain) was obtained from the American Type Culture Collection (ATCC) (catalog#VR-30, ATCC, Manassas, Virginia, USA) and grown in Vero cells (catalog# CCL-81, ATCC, Manassas, Virginia, USA) to create a virus stock (16). 100 μL of tissue culture-passaged virus stock (10^3^ PFU) was injected ip into 4-week-old female BALB/c mice (strain #000651, Jackson Laboratory, Bar Harbor, Maine, USA) and virus was obtained from hearts at day 3 pi by homogenization in Gibco Minimum Essential Media (cat#11095-080, Thermo-Scientific, Waltham, Massachusetts, USA) supplemented with 2% heat inactivated fetal bovine serum (16). Heart homogenate was centrifuged at 4°C for 20 minutes at 795 *g* (16). Supernatant, which contains infectious virus and damaged heart proteins (termed heart-passaged virus) was stored at -80°C until used to induce myocarditis (16). 0.25 mL of PEV, pmPEV, or 1x PBS vehicle control was administered ip to mice on days -1, 0, 1 with 10^3^ PFU of heart-passaged virus injected ip on day 0 and myocarditis examined at day 10 pi.

### 2.5 Histology

Mouse hearts were cut in half longitudinally, fixed in 10% phosphate-buffered formalin for 48 hours, and embedded in paraffin, as previously reported (28). 5 µm sections were used for all histological staining. Hematoxylin and eosin (H&E) stain was used to assess myocarditis and pericarditis, or the percentage of the heart with myocardial or pericardial inflammation, respectively, normalized to the overall size of the heart section, as previously described (28–30). Masson’s trichrome was used to assess fibrosis, or collagen deposition, normalized to the total size of the heart section. CD45 (Biolegend, San Diego, CA, 103102, 1:200, rat), CD11b (Abcam, Cambridge, United Kingdom, ab133357, 1:3000, rabbit), CD3 (Abcam, ab16669, 1:200, rabbit), F4/80 (BioRad, Hercules, CA, MCA497G, 1:250, rat), TLR4 (Novus Biologicals, Littleton, CO, NB100-56580-0.1mg, 1:400, rabbit), TLR2 (Abcam, Cambridge United Kingdom, ab209216, 1:200, rabbit), or C3aR (R&D, Minneapolis, MN, MAB10417-100, 1:200, rat) with secondary antibodies (anti-rabbit: cat#K4003, Envision+ anti-rabbit labeled polymer, Agilent (Dako), Santa Clara, CA or cat#RT517 rat-on-rodent kit, Biocare, Pacheco, California, USA) were used to assess specific immune cell populations in the heart. Stained slides were scanned using an Aperio AT2 slide scanner to select representative images (Leica, Wetzlar, Germany). To analyze immunohistochemistry slides, the ventricles of each sample were manually selected by a lab member blinded to study groups. The default “positive pixel” algorithm from Aperio eSlide Manager (Leica, Wetzlar, Germany) was modified for each stain by adjusting the Color Saturation Threshold so that the program’s selection of positive and negative pixel counts accurately reflected each stain with hue set at 0.1 or brown (CD45: 0.03, CD11b: 0.14, CD3: 0.03, F4/80: n/a, TLR4: 0.10, TLR2: 0.03, C3aR: n/a), as previously described (12). The percentage of positive pixels within each annotation was determined with a positivity parameter as a surrogate for stain positivity: positivity = number of positive pixels/(total number of pixels, both positive and negative, in the annotation layer), as previously described (31).

### 2.6 Quantitative real-time PCR

RNA isolation was performed on heart tissue, which was first homogenized using a Tissuelyser (Qiagen, Germantown, Maryland, USA), with 7 mm stainless steel beads in RNeasy Lysis Buffer (RLT) with 0.5% DX buffer to reduce foam, as previously (32). A QIAcube instrument (catalog#9001292, Qiagen, Germantown, Maryland, USA) was used to automatically isolate and purify RNA, with reagents for RNase easy fibrous mini kit including a DNase and proteinase K step (catalog#74704, Qiagen, Germantown, Maryland, USA). RNA quantification was determined in µg/µL using NanoDrop (Thermo Scientific, Waltham, MA), as previously described (29, 32, 33). RNA was converted to complementary DNA (cDNA) using high-capacity cDNA reverse transcriptase kit (catalog#4368813, Applied Biosystems, Foster City, California, USA), as previously described (29, 32, 33). Gene expression from mouse hearts was assessed by quantitative real-time polymerase chain reaction (qRT-PCR) using assay-on-demand primers and probe sets and the ABI 7000 Taqman system (Applied Biosystems, Foster City, California, USA). Probe sets listed in **Supplementary Table 2** were purchased from Thermo-Scientific (Waltham, Massachusetts, USA). Probe sets to detect CVB3 viral protein 1 (VP1) were obtained from Integrated DNA Technologies (Coralville, Iowa, USA) (34). Gene expression was analyzed by assessing comparative quantification, which is then used to calculate relative gene expression (RGE) using the formula: RGE = 2 − (ΔC_t_ − ΔC_t_(max)), as previously.

### 2.7 Protein sequencing and analysis

Trypsin digestion and column fractionation were performed on reconstituted samples prior to tandem liquid chromatography with tandem mass spectrometry, as detailed below. The Mayo Clinic Proteomic Analysis CORE completed primary and secondary analyses.

#### 2.7.1 Tandem mass tag labeling of peptides

100μg of peptide from each sample was solubilized in 100μL of 100mM triethylammonium bicarbonate (TEAB), pH 8.5, and mixed with 100μg of a unique TMT 6plex reagent solubilized in 10μL of acetonitrile. After incubation for 1h at room temperature the reactions were quenched with 5μL of 5% hydroxylamine and a 2μL aliquot from each sample was pooled and analyzed by tandem mass spectrometry to ensure the labeling efficiency was greater than 98%. The samples were then pooled to match the reporter ion intensities from each channel and the mix was acidified. Excess TMT reagents were removed using solid phase extraction with a Waters Sep Pak Plus C18 cartridge and the eluted TMT labeled peptides lyophilized.

#### 2.7.2 Basic pH HPLC fractionation

To reduce the sample complexity, the dried peptide mixture was solubilized in 500μL 10mM ammonium formate, pH 8.5 and separated into 96 fractions using a Dionex Ultimate 3000 RS HPLC system with a Waters XBridge BEH C18 4.6mm x 250mm column. The system was set up with 10mM ammonium formate; pH 8.5 in water for the A solvent and 10mM ammonium formate; pH 8.5/90% acetonitrile for the B solvent. The separation gradient was 5%B to 60%B over 60 minutes followed by a 2-min jump to 80%B while maintaining a constant flow rate of 0.5ml/minute. The 96 fractions were concatenated to 24 fractions and lyophilized.

#### 2.7.3 NanoLC-tandem mass spectrometry data acquisition

The peptide fractions were analyzed by nanoLC-tandem mass spectrometry using a Thermo Scientific Exploris 480 Orbitrap mass spectrometer coupled to a Thermo Ulimate 3000 RSLCnano HLPC system with 0.1% formic acid in 98% water/2% acetonitrile for the A solvent and 0.1% formic acid in 80% acetonitrile/10% isopropanol/10% water for the B solvent. Each fraction was solubilized in 0.1% formic acid and pumped onto a Halo C18 2.7µm EXP stem trap (Optimize Technologies, Oregon City, OR) with 0.1% formic acid/0.05% TFA at a flow rate of 8mL/minute. The trap was placed in line with a 50cm x 75um EasySpray C18 column and the peptides separated with a gradient of 3%B to 35%B over 90minutes at a flow rate of 300nL/minute. The mass spectrometer was set for data dependent acquisition with a 3 sec cycle time. The MS1 survey scan range was from 350-1600 m/z at resolution 120,000 (at 200m/z) and the AGC set for a maximum of 1E6 ions and a 50ms ion injection time. Ions in the scan range of 350-1600 m/z with positive charge states from 2-4 were sequentially selected for high energy collisional dissociation (HCD) fragmentation MS/MS scans at resolution 45,000 with a NCE setting of 39 and the isolation width set to 0.7 m/z. The MS2 AGC setting was 200% (2E5 ions) and the max ion injection time was set to 105ms. The dynamic exclusion feature was used to prevent ions selected for MS2 and any ions within an m/z of 7ppm from being selected for fragmentation for 30 seconds.

#### 2.7.4 Protein identification and TMT quantitation

The mass spectrometry raw data files were analyzed using Proteome Discoverer 2.5 (Thermo Scientific), setup for MS2 reporter ion quantification with TMT 6plex isobaric labels. The fractions were searched against a Swissprot human (2020_01) database using Sequest HT with parameters set for full trypsin specificity with oxidized Met and N-term protein acetylation allowed as variable modifications and carbamidomethyl cysteine, TMT lysine and TMT peptide n-terminus as fixed modifications. Mass tolerances were set at 10 ppm for precursor ions and.02 Dalton for MS2 fragment ions. Protein identifications with a 1 peptide minimum were filtered at 1% FDR using the Percolator node. Reporter ion channel correction factors were applied to PSMs and filtered to exclude peptides exceeding the threshold maximum of 50% isolation interference. Sample groups missing values were removed for comparisons with no imputation applied.

### 2.8 Proteomic pathway enrichment visualization

We performed pathway enrichment analysis to create multiple visualizations of our data using minor modifications to the protocol presented by Reimand et al. (35).

To visualize the differentially expressed proteins between PEV and pmPEV, we performed enrichment analysis on all transcripts with nominal p-value < 0.01. Gprofiler (36) was used to run an ordered query on this list, which was ordered from most to least significant p value beneath this cut off. Duplicates or transcripts without a known Ensembl ID were excluded to clean this list and the ordered query was rerun on the cleaned data, with the term size limited to 5-350, as per recommendation (35). Cytoscape V8.0.0 was used to visualize enriched pathways of this dataset with the output files from gProfiler and the Enrichment Map application within this software. Node cutoff was set to FDR(Q) value < 0.01 and default edge cutoff was used. Nodes were colored based on log fold change value and manually arranged to allow easy visualization of differences between the products.

We were also interested in displaying the distinctly enriched proteomes of both the PEV and pmPEV products and used the same methods to display these data for transcripts with p values < 0.01 of positive log2-fold change (PEV) or negative log2-fold change (pmPEV). After gProfiler analysis and Enrichment Map visualization, the nodes were further ‘super’ clustered based on their shared genes and the AutoAnnotate function within Cytoscape/Enrichment Map was used to identify clusters of nodes with shortened pathway names.

Finally, we wanted to display overlapping pathway involvement of 10 proteins shared by our 2 products and 3 similar plasma/platelet EV products from the literature (37–39). We used Gprofiler to run a non-ordered query on this list, with the same term size limits as above, and then used Cytoscape to visualize shared pathways of these proteins. AutoAnnotate was again used to label superclusters of proteins.

### 2.9 MicroRNA sequencing and analysis

Next-generation sequencing for miRs was performed by the Mayo Clinic Molecular Biology Core. Three samples of each product (PEV and pmPEV) were resuspended and analyzed. Qubit fluorometry (ThermoFisher Scientific, Waltham, Massachusetts, USA) and an Agilent BioAnalyzer (Santa Clara, California, USA) were used to determine total RNA concentration and quality. RNA libraries were prepared from 1 ng total RNA according to Qiagen’s guidelines for the QIAseq miRNA Library Kit (Qiagen, Germantown, Maryland, USA). Briefly, adaptors were ligated to the 3’ and 5’ ends of miRs, then a complementary primer was annealed to 3’ adaptor sequences followed by reverse transcription to generate a cDNA library of small RNAs. cDNA libraries were purified and then enriched, where a unique index was added to each sample. A final purification step was performed prior to the quantitation of completed libraries. Libraries were sequenced at approximately 30 million read pairs per sample, following Illumina’s standard protocol using the Illumina cBot (catalog#SY-301-2002, San Diego, California, USA) and HiSeq 3000/4000 PE Cluster Kit (catalog#PE-410-1001, Illumina, San Diego, California, USA). The flow cell was sequenced as 50 X 2 paired end reads on an Illumina HiSeq 4000 using HiSeq 3000/4000 sequencing kit and HD 3.4.0.38 collection software. Base-calling was performed using Illumina’s RTA version 2.7.7.

### 2.10 Sequencing visualization

R analysis was used to visualize sequencing results in heat maps and principal component analysis (PCA) plots. Heatmaps were created using Pheatmap version 1.0.12 within R 4.3.1 using row-wise scaling to ensure data normalization, followed by the application of unbiased hierarchical clustering for both rows and columns. This allowed identification of inherent patterns, groupings, and outliers within the data, facilitating comprehensive data exploration. PCA plots were created using the ‘stats’ package in R 4.3.1. This allowed calculation of the explained variance and the contribution of each principal component to the overall variance. Subsequently, ‘dplyr’ version 1.1.2 and ‘plotly’ version 4.10.2 were used to visualize PCA results in a three-dimensional plot. PC1 was represented on the X-axis, PC2 on the Y-axis, and PC3 on the Z-axis, offering a comprehensive view of the data’s underlying structure.

### 2.11 Statistical analysis

Normally distributed data comparing three groups were analyzed using a one-way analysis of variance (ANOVA) and Dunnett’s test for multiple comparisons, unless otherwise specified. Statistical outliers were identified in Prism and removed prior to analysis. The data were expressed as scatter plot and mean ± standard error of the mean (SEM), unless otherwise noted. A value of *p* < 0.05 was considered significant. Precise *p* values for comparisons are listed in the text while graphs display *, *p* < 0.05; **, *p* < 0.01; ***, *p* < 0.001 and ****, *p* < 0.0001. Statistical analyses were performed using GraphPad Prism 9.5.1.

## 3 Results

### 3.1 PEV and pmPEV display classic EV characteristics

**Figure 1:**
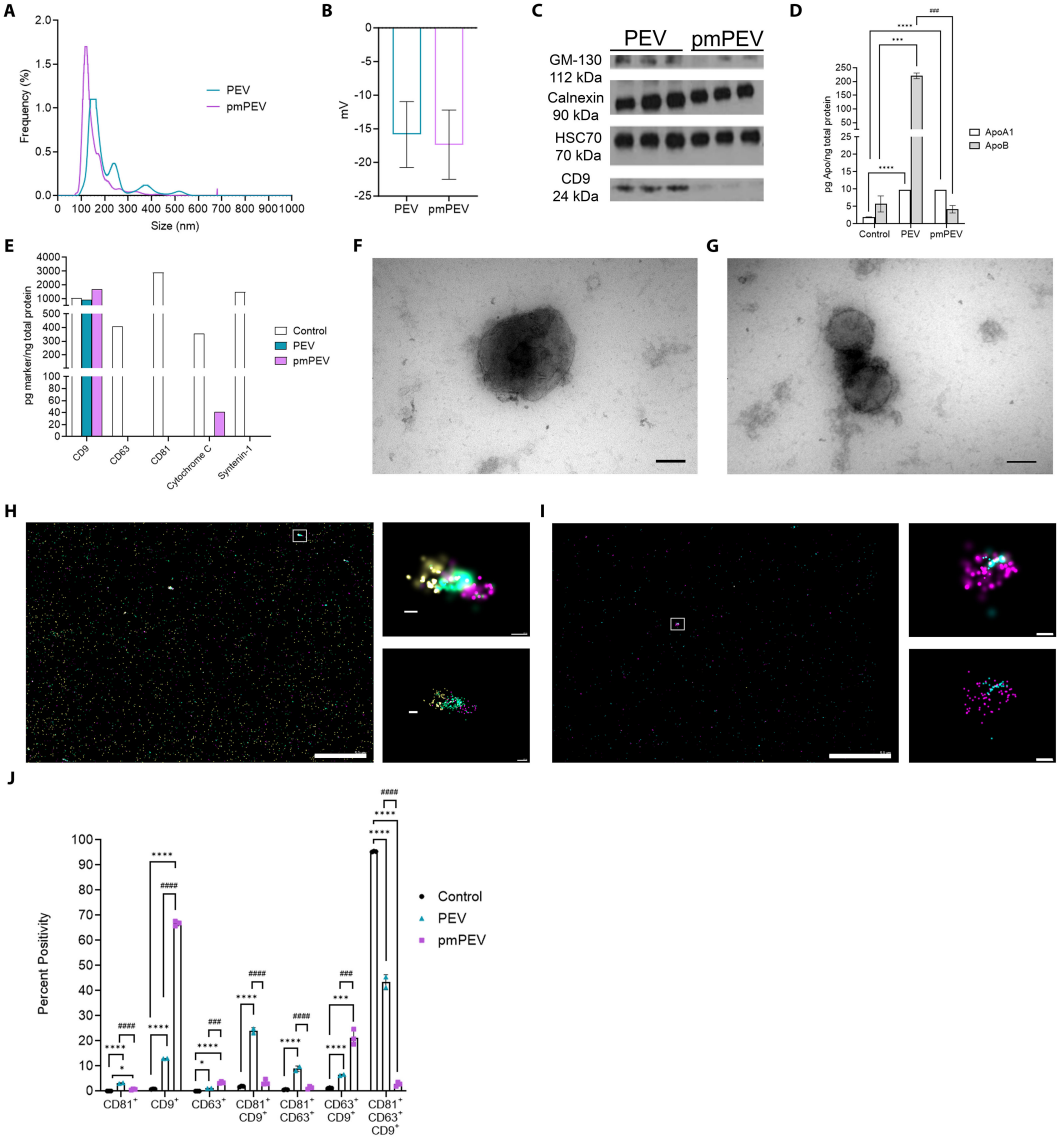
Characterization revealed platelet-derived EVs (PEV) and premenopausal (pm)PEV display typical extracellular vesicle (EV) profiles. PEV and pmPEV were reconstituted at 11.2 mg/mL or 8.0 mg/mL, respectively, as per manufacturer instructions. For characterization, size-exclusion chromatography (SEC) was performed on samples to provide a smaller noise to particle ratio. Fractions 7-11 from SEC were analyzed for size distribution using **(A)** nanoparticle tracking analysis, which was also measured by tunable resistive pulse sensing (TRPS) in **Supplementary Figures 1C–E**. TRPS was also used to measure **(B)** zeta potential, shown as mean ± SD. **(C)** Western blot analysis was performed to test for positive and negative EV markers, full blots found in **Supplementary Figures 1A, B**. **(D)** ELISAs and **(E)** EV multiplex assays were performed on samples in duplicate after reconstitution and without SEC to appropriately estimate relative abundance of positive and negative markers of EVs. **(F, G)** PEV and pmPEV samples were visualized with transmission electron microscopy for morphology (magnification 150,000X, *Scale bars*: 100 nm); widefield images found in **Supplementary Figures 1F–I**. Visualization was also conducted with directed stochastic optical reconstruction microscopy (dSTORM) for tetraspanin profiling; CD9 (yellow), CD81 (teal), CD63 (magenta). **(H, I)** Large left image and right top image show raw depictions and right bottom image shows precision depictions of dSTORM; *Scale bars*: left image 5.0 µm; top and bottom images 100 nm. **(J)** Quantification of tetraspanin profiling using percent positivity was measured for each marker (single, double, or triple positive EVs) and for each sample. **(J)** Data show mean ± SD quantified from 3 independent pictures for each sample. Statistical analysis performed by one-way analysis of variance (ANOVA) with Tukey’s multiple comparison test; *, *p* < 0.05; ***, *p* < 0.001; ****, *p* < 0.0001 compared to control. ###, *p* <  0.001; ####, *p* <  0.0001 compared to PEV.

PEV and pmPEV were reconstituted from a lyophilized powder with PBS prior to characterization, as previously described (12, 24, 25). Size distribution using nanoparticle tracking analysis (NTA) showed that the mean diameter of PEV was 208.2 ± 13.3 nm and pmPEV was 155.1 ± 3.5 nm, with two modal peaks at 100-200 nm and 200-300 nm for PEV versus one modal peak at 90-160 nm for pmPEV (**Figure 1A**). These findings were confirmed using the orthogonal technique tunable resistive pulse sensing (TRPS), showing a mild secondary peak for PEV and only one model peak for pmPEV at similar approximate sizes to the NTA analysis (**Supplementary Figures 1C–E**). TRPS also revealed an expected negative zeta potential charge for both products (**Figure 1B**) (40, 41). Western blot identified the presence of classic EV markers including cytosolic heat shock cognate protein 70 (HSC70) and the membrane tetraspanin CD9; however, the presence of negative EV markers calnexin and Golgi matrix protein 130 (GM-130) were also detected in both products (**Figure 1C**) confirming the presence of both EVs and intracellular nanoparticles in these heterogenous blood platelet products. Full images of the western blots are found in **Supplementary Figures 1A, B**. ELISAs of ApoA1 and ApoB showed that PEV had higher ApoB content than pmPEV while PEV and pmPEV contained similar amounts of ApoA1 (**Figure 1D**) indicating the presence of Apo in both products. A reference EV control from HCT116, a human cancer colorectal cell line, was also included to allow comparison of these products to a commercially available, standardized EV product. The control also contained ApoA1 and ApoB demonstrating that even ‘purified’ controls contain similar co-isolating, non-EV contaminants (**Figure 1D**). A multiplex EV assay for five markers of EVs quantitated abundance of tetraspanins CD9, CD63, and CD81, and cytosolic proteins cytochrome C and syntenin-1 in each product compared to the EV control (**Figure 1E**).

Using an EV marker multiplex assay kit the standardized control showed the presence of all EV markers, while PEV contained only CD9 and pmPEV contained CD9 and cytochrome c (**Figure 1E**). Cytochrome c is associated with mitochondria and can be found in both the cytoplasm and in EVs, although this marker is not specific to EVs and may be found in intracellular vesicles as well, perhaps demonstrating either pathological processes in the cell concerning mitochondria or contamination of the EV control and pmPEV product with intracellular particles (3). Transmission electron microscopy revealed a heterogenous mixture of membrane-bound particles with internal electron densities and spherical morphology consistent with the expected shape of EVs as well as background debris and clumping of particles, suggesting the presence of lipoproteins and other non-EV components (**Figures 1F, G** and **Supplementary Figures 1F–I**). Direct stochastic optical reconstruction microscopy (dSTORM), a super resolution microscopy capable of localizing single molecules at a resolution of 20 nanometers, was used to visualize individual EVs in 3D space as well as visualizing tricolor antibody staining of the tetraspanins CD81, CD9, and CD63 (**Figures 1H, I**). Although CD63 and CD81 were not detected in bulk multiplex assay analysis, the highly sensitive dSTORM technique allowed detection of all three tetraspanins in PEV and pmPEV products as well as in the EV control (**Figure 1E** versus **Figures 1H, I**). Quantification of dSTORM revealed that PEV had relatively more CD81^+^/CD9^+^/CD63^+^ triple positive EVs, while pmPEV had more CD9^+^ single positive EVs (**Figure 1J**). Additionally, PEV had more CD81^+^/CD9^+^ and CD81^+^/CD63^+^ double positive EVs while pmPEV had more CD63^+^/CD9^+^ double positive EVs (**Figure 1J**). This heterogeneity is typical for EVs and the significance of the observed tetraspanin distributions is currently unclear. Overall, these findings indicate that PEV and pmPEV share typical EV characteristics and are heterogenous products that also contain non-vesicular nanoparticles.

### 3.2 PEV and pmPEV treatments decreased acute myocarditis, but PEV was more effective

We recently published that innate treatment of male BALB/c mice with PEV significantly decreased myocardial inflammation and fibrosis and improved cardiac function (12). In this study we wanted to determine whether donor factors of sex and age altered the efficacy of two different EV products. We hypothesized that pmPEV from young females would be more effective than PEV from males and females of all ages in preventing myocarditis. This rationale was based on the known protective effect of estrogen in reducing cardiovascular disease in women, the lower incidence of myocarditis in women, and preclinical research showing that estrogen reduces myocardial inflammation in female mice with CVB3 myocarditis (14, 19, 42).

**Figure 2:**
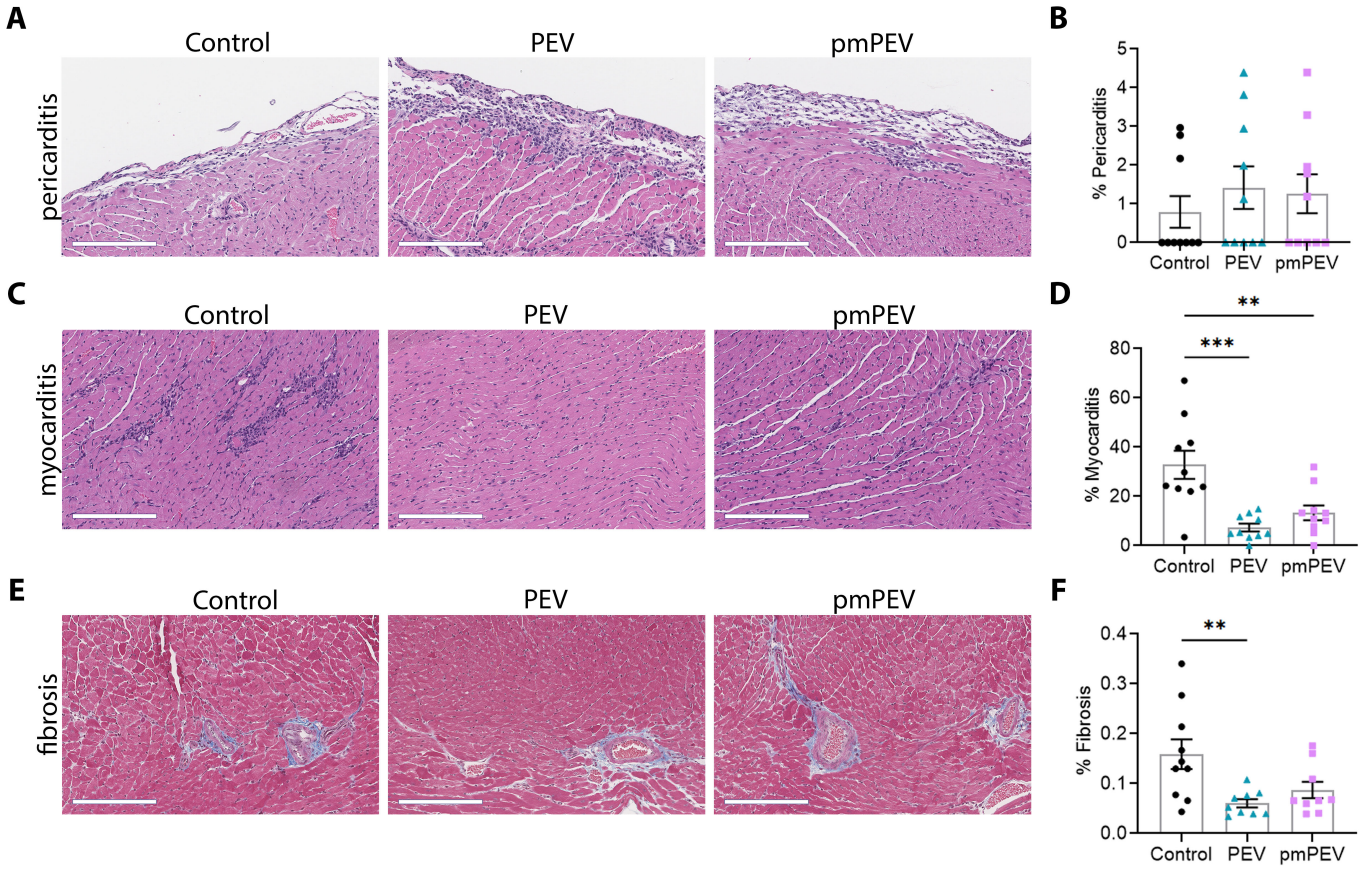
Treatment with PEV or pmPEV decreased myocardial inflammation and fibrosis. EVs from healthy men and women (PEV), or from healthy premenopausal women (pmPEV) (0.25 mL of 10^10^ EVs/mL), or control (0.25 mL of 1X PBS) were given ip to male BALB/c 8-week-old mice on days -1, 0, 1 pi with 10^3^ PFU of CVB3 given ip on day 0. Representative photos of **(A)** pericarditis, **(C)** myocarditis, **(E)** fibrosis at day 10 pi; *Scale bars*: 200 µm. Quantification of inflammation **(B, D)** or fibrosis **(E)** was performed on individual hearts. Data show mean ± SEM for 10 mice/group, one-way ANOVA with Dunnett’s multiple comparison, *, *p* < 0.05; **, *p* < 0.01; ***, *p* < 0.001 (see **Supplementary Table 3**).

In this study we treated male BALB/c mice with 250 μL of PEV or pmPEV or PBS vehicle control injected intraperitoneally (ip) on days -1, 0, and +1 post infection (pi), while CVB3 with heart proteins was also injected ip on day 0 and we assessed endpoints during peak acute myocarditis at day 10 pi, as previously (12). Clinically myocarditis often presents as perimyocarditis (43), which is also found in our model of CVB3 viral myocarditis (12, 33, 44, 45). For that reason, we assessed the effects of PEV or pmPEV treatments on myocarditis (that is, perimyocarditis) and pericarditis (that is, pericardial inflammation alone) using H&E staining (**Figures 2A–D**). We found that neither PEV nor pmPEV significantly altered pericarditis compared to PBS treated controls (*p* = 0.64, *p* = 0.78, respectively) (**Figures 2A, B**; **Supplementary Table 3**). However, both PEV (*p* = 0.0002) and pmPEV (*p* = 0.0036) significantly reduced myocarditis (**Figures 2C, D**; **Supplementary Table 3**). There were no significant differences comparing PEV to pmPEV for myocarditis (*p* = 0.52) or pericarditis (*p* = 0.97) (**Figures 2B, D**; **Supplementary Table 3**). During acute myocarditis, fibrosis may be observed at this early stage of the pathogenesis of disease primarily around vessels with very little, if any, myocardial fibrosis (44, 46). Quantifying scans of slides stained with trichrome blue to detect collagen deposition revealed that PEV significantly reduced fibrosis/perivascular fibrosis compared to PBS treated controls (*p* = 0.0079) at this acute myocarditis timepoint, which was not observed for mice treated with pmPEV compared to controls (*p* = 0.0601) (**Figures 2E, F**; **Supplementary Table 3**). In our prior work, we saw that decreased fibrosis at day 10 pi corresponded with prevention of cardiac dysfunction later during chronic myocarditis (12), suggesting that PEV may have greater potential than pmPEV to prevent progression to chronic myocarditis/DCM.

To further confirm these findings, we conducted immunohistochemistry (IHC) to verify protein levels of key immune cell and signaling markers (**Supplementary Figure 2**; **Supplementary Table 4**). Heart sections were stained individually for four key markers of immune cells including CD45 (all lymphocytes), CD11b (activated macrophages, mast cells, neutrophils, and some dendritic cells), CD3 (all T cells), and F4/80 (macrophages), TLR4, TLR2, and one marker of the complement cascade C3aR (**Supplementary Figure 2**). We found that PEV reduced most of these markers during myocarditis, including CD45 (*p* = 0.0187), as well as CD11b (*p* = 0.0305), CD3 (*p* = 0.0087), F4/80 (*p* = 0.0177), and C3aR (*p* = 0.0007) compared to the PBS-treated control, while pmPEV only reduced C3aR (*p* = 0.0157) (**Supplementary Figure 2**; **Supplementary Table 4**). While neither treatment significantly reduced TLR4 or TLR2 compared to control, PEV reduced TLR2 more than pmPEV (*p* = 0.0489) (**Supplementary Figure 2**; **Supplementary Table 4**). These IHC data reinforce a pattern of PEV eliciting stronger immune regulation effects than pmPEV.

### 3.3 Global immune gene downregulation occurred with PEV and pmPEV treatment during myocarditis, but was more comprehensive for PEV

**Figure 3:**
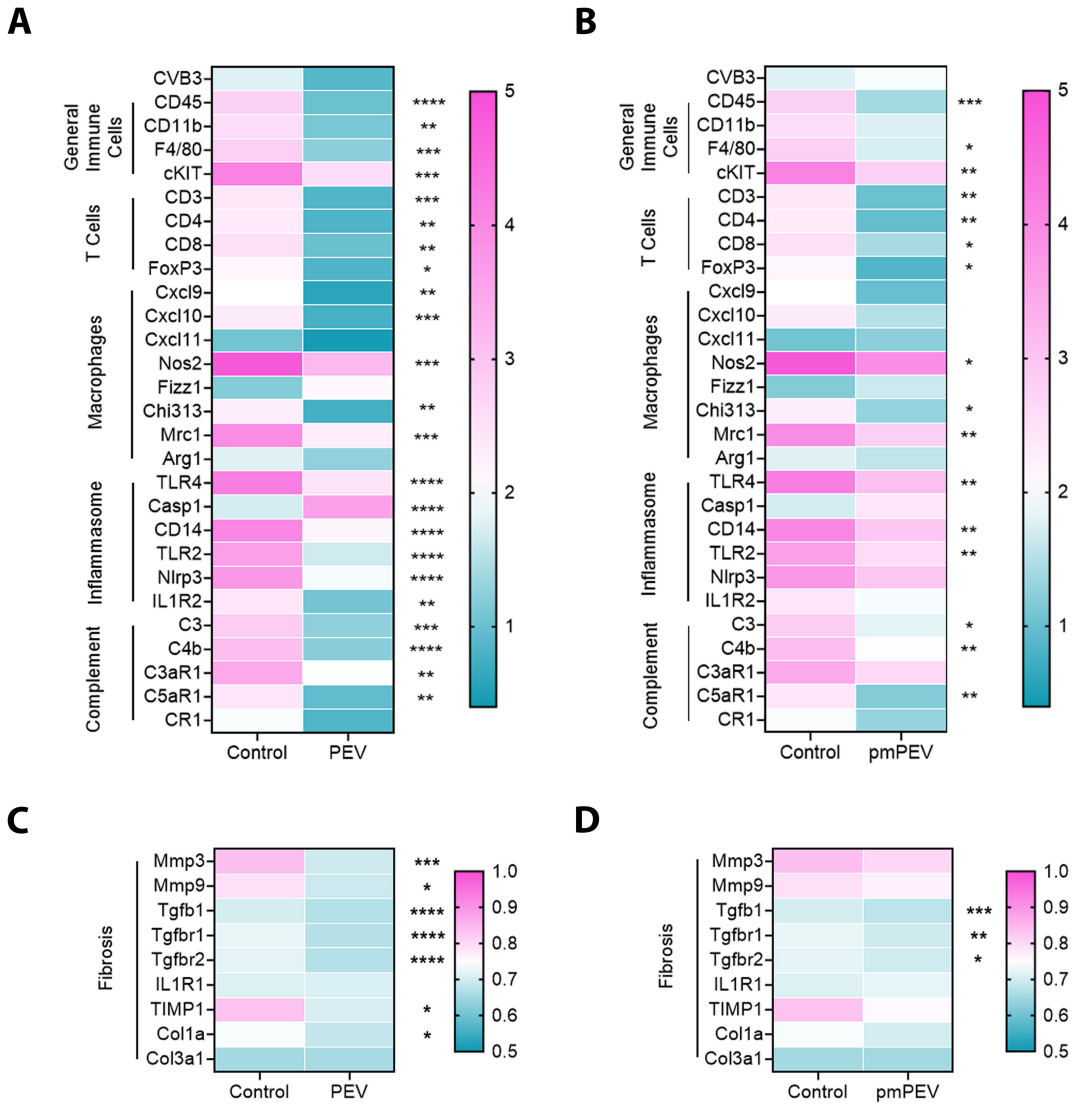
PEV and pmPEV globally downregulate immune and fibrosis markers, but more changes occur with PEV. EVs from healthy men and women (PEV), or from healthy premenopausal women (pmPEV) (0.25 mL of 10^10^ NPs/mL) or control (0.25 mL of 1X PBS) were given to male BALB/c 8-week-old mice ip on days -1, 0, 1 pi with 10^3^ PFU of CVB3 given ip on day 0. Relative gene expression (RGE) from whole hearts by qRT-PCR at day 10 pi was used to assess **(A, B)** acute myocarditis immune markers and **(C, D)** fibrosis markers indicated on the left axis of each matrix in individual experiments compared to the housekeeping gene *Hprt* (see **Supplementary Figure 3** for individual data). Data show mean RGE for 10 mice/group, one-way ANOVA with Dunnett’s multiple comparison were conducted for each marker but are depicted separately as PEV or pmPEV vs. control; *, *p* < 0.05; **, *p* < 0.01; ***, *p* < 0.001; ****, *p* < 0.0001 (see **Supplementary Figure 3**, **Supplementary Table 5**).

As mentioned earlier, the immune profile that increases CVB3 myocarditis in male mice at day 10 pi has been extensively studied and is known to involve several key pathways including activation of both mast cells and macrophages by complement, TLR2, TLR4/IL-1 receptor, and inflammasome pathways that lead to elevated IL-1β levels and increased remodeling/fibrosis. We examined these primary immune cells and signaling pathways using quantitative real time polymerase chain reaction (qRT-PCR) to compare the treatment groups to controls (**Figure 3**). Results shown in the gene matrices in **Figure 3** are displayed individually by gene in **Supplementary Figure 3** and individual ANOVA and pairwise comparisons for each gene are shown in **Supplementary Table 5**. Importantly, qRT-PCR revealed that treatment with PEV (*p* = 0.12) or pmPEV (*p* = 0.74) did not alter CVB3 viral protein levels in the heart compared to PBS treated mice during myocarditis (1-way ANOVA *p* = 0.14) (**Figures 3A, B**). We found that both PEV and pmPEV significantly decreased most of the immune genes that we examined, confirming histology findings. However, more genes were decreased by PEV treatment that were not decreased by pmPEV treatment, including *CD11b* (*p* = 0.0018), C-X-C motif chemokine ligand (Cxcl)-9 (*p* = 0.0047), *Cxcl10* (*p* = 0.0009), caspase-1 (*Casp1*, *p* = 0.0018), nucleotide-binding domain, leucine-rich-containing family, pyrin domain-containing-3 (*Nlrp3*) (*p* = 0.0075), interleukin (IL)-1 receptor 2/*ST2* (*p* = 0.0076), and complement component 3 antagonist receptor 1 (*C3aR1*) (*p* = 0.0029) (**Figures 3A, B**; **Supplementary Figure 3**; **Supplementary Table 5**). Additionally, PEV reduced fibrosis markers, which in contrast remained unchanged after pmPEV treatment, including matrix metalloprotease (*Mmp)3* (*p* = 0.0032), *Mmp9* (*p* = 0.0391), tissue inhibitor of metalloproteinases 1 (*TIMP1*) (*p* = 0.0134) and collagen 1A1 (*Col1a1*, *p* = 0.0415) (**Figures 3C, D**; **Supplementary Figure 3**; **Supplementary Table 5**). Overall, analysis of genes known to be important in contributing to CVB3 myocarditis in males showed more global reduction using PEV treatment compared to pmPEV treatment, which was the opposite of our original hypothesis.

### 3.4 PEV and pmPEV display distinct proteomic profiles

**Figure 4:**
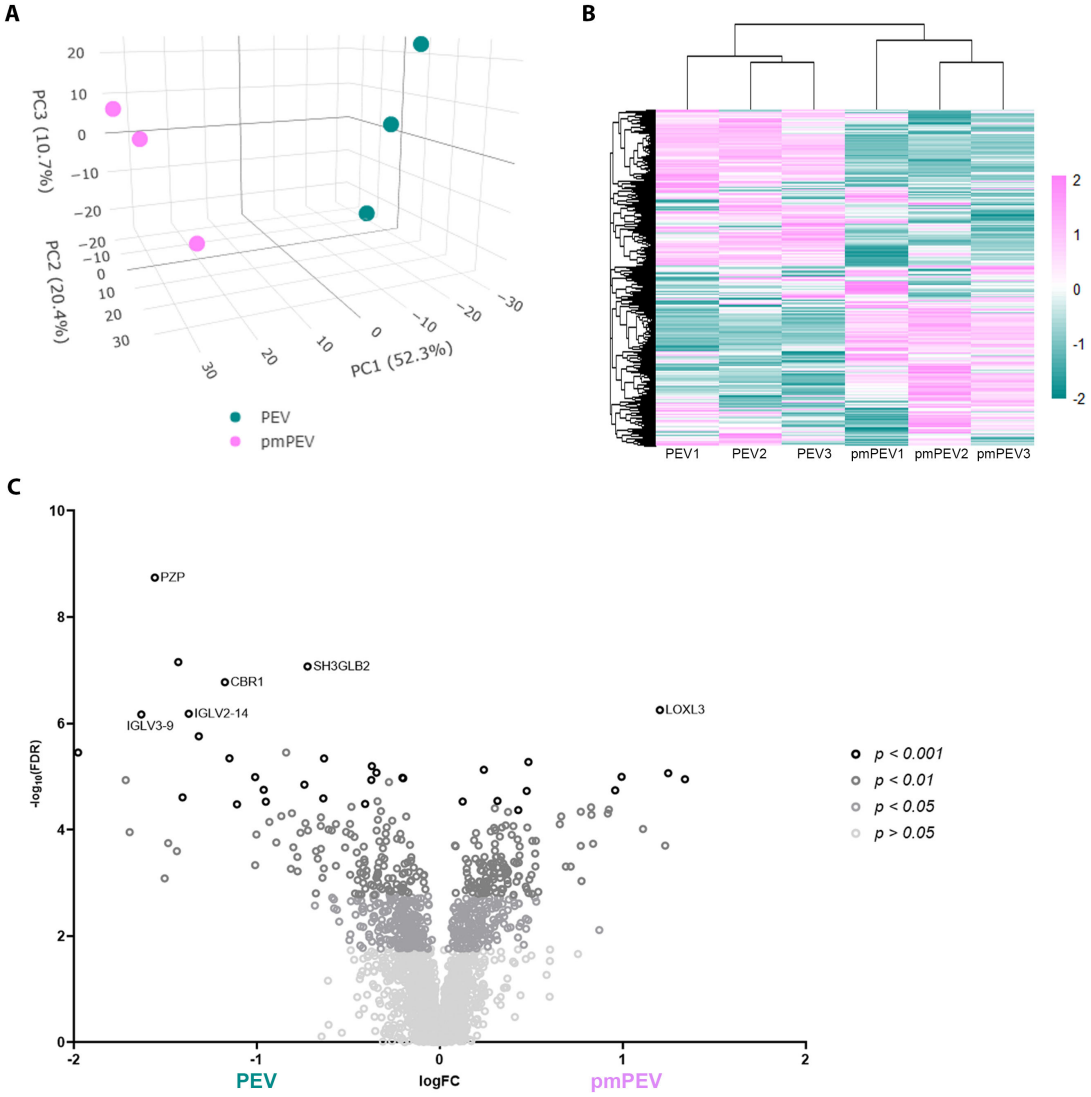
Initial assessment of PEV versus pmPEV proteome. **(A)** Principal component analysis. **(B)** Hierarchal clustering. **(C)** Volcano plot of significantly different proteins.

To better understand the contents of PEV and pmPEV, we performed proteomic analysis of each product. Principal component analysis of these proteomes revealed that PEV and pmPEV displayed distinct protein profiles with product replicates grouping together (**Figure 4A**). Non-biased hierarchal clustering further confirmed that the EV products grouped tightly within their own replicates, yet PEV and pmPEV were clearly distinct products (**Figure 4B**). A volcano plot of comparisons of individual protein expression in PEV versus pmPEV showed a large, shared proteome (*p* > 0.05), but also many proteins that were significantly different between PEV and pmPEV (**Figure 4C**). The most differently expressed proteins (*p* < 0.001) in PEV were pregnancy zone protein (PZP) (α2-macroglobulin protease inhibiter associated with pregnancy (47)], cytochrome b5 reductase 1 (CBR1) [a NADPH-dependent oxidoreductase important in ATP production found to decrease oxidative stress and inflammation (48, 49)], endophilin-β2 (SH3GLB2) [elevated after viral infection, associated with autophagy (50, 51)], and immunoglobulins (Ig) V2-14 and V3-9 (**Figure 4C**). In contrast, the most differentially expressed protein in pmPEV (*p* < 0.001) was lysl-oxidase L3 (LOXL3) [important in crosslinking collagen and elastin increasing T cell-associated fibrosis and cardiomyopathy (52–54)] (**Figure 4C**).

### 3.5 Proteomic analysis reveals immune-specific enrichment in PEV and pmPEV

**Figure 5:**
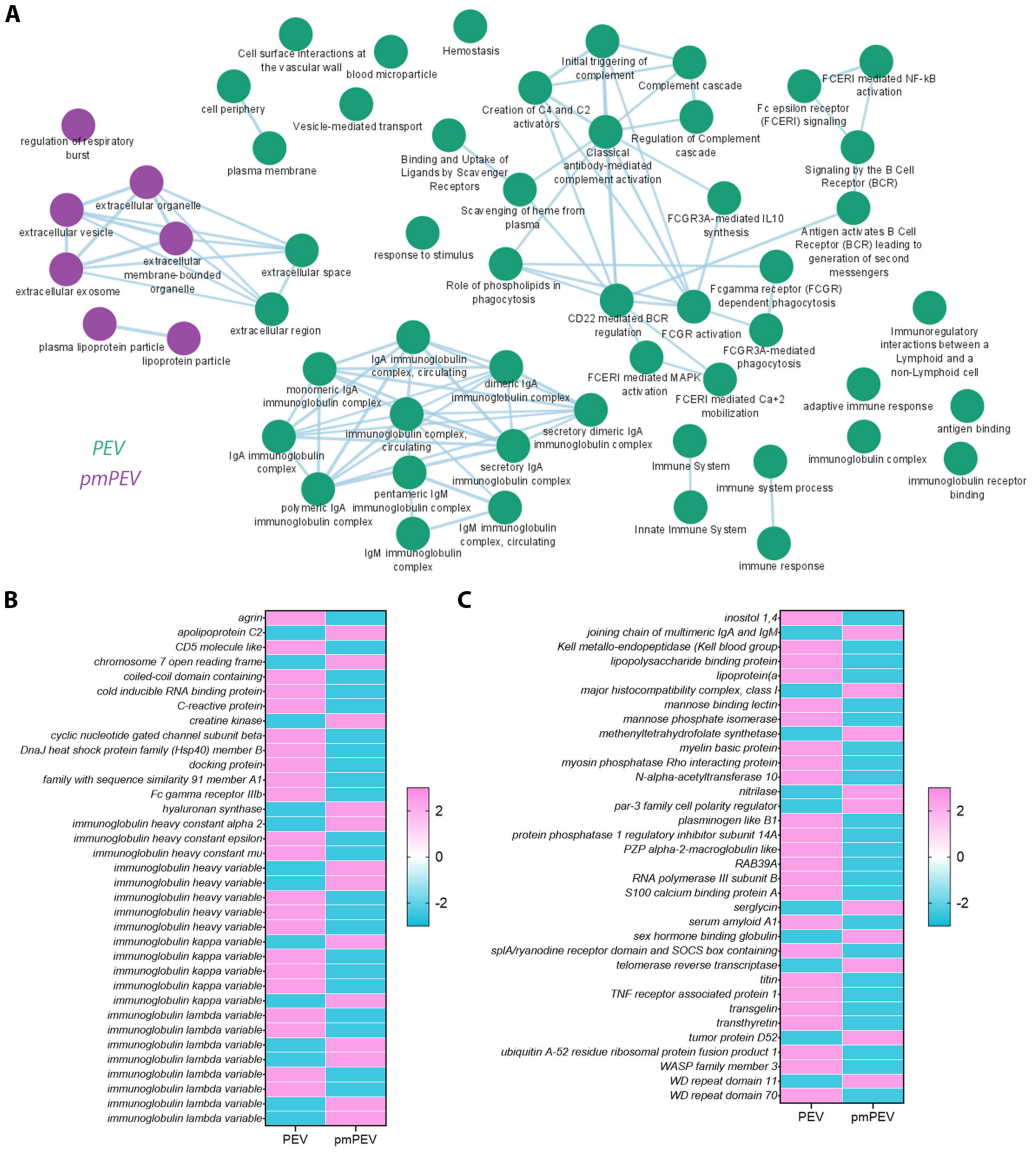
Proteomic analysis of PEV versus pmPEV reveal immune-specific enrichment in PEV and pmPEV. **(A)** Cytoscape pathway shows super-clusters of proteins in PEV (green) versus pmPEV (purple). Differentially enriched proteins between **(B)** PEV and **(C)** pmPEV with adjusted *p* value < 0.01.

To better understand the proteins that were differentially expressed in PEV versus pmPEV, we applied a pathway enrichment pipeline to the proteome data which identified that, in general, more immune pathways were enriched in PEV while pmPEV was mainly enriched for general EV or biogenic nanoparticle pathways (**Figure 5A**). However, both products were enriched for various proteins associated with EVs or non-vesicular particles, and proteins involved in increasing inflammation (**Figures 5B, C**).

Although some proteins in each product were reported to be immunoregulatory in the literature, the majority of the proteins differentially expressed in PEV or pmPEV were associated with promoting inflammation (**Tables 1**, **2**, respectively). Many immunoglobulins/immunoglobulin (Ig) components were increased in PEV and pmPEV including IgE and IgG, which may have activated/maintained activation of pericardial mast cells providing at least one possible explanation for why PEV and pmPEV treatment did not reduce pericardial inflammation which is associated with mast cell degranulation (74–76). Although PEV and pmPEV had many distinct proteins (**Tables 1**, **2**), the overall functions of most of these proteins from both products was similar in that they may promote inflammation, based on the literature.

**Table 1:** Roles of differentially expressed proteins in PEV according to the literature.

Protein	Protein definition	Log2fold change	Roles in literature	References
HAS1*^a^*	Hyaluronan synthase 1	-3.27	Polysaccharide in extracellular matrix associated with inflammation and remodeling	(55)
KEL	Kell blood group glycoprotein	-1.98	Important in transfusion medicine because immunogenic and alloantibodies are often produced when unmatched blood is transfused	
FAM91A1	Protein FAM91A1	-1.72	Golgi-mediated capture of vesicles generated using AP-1	
TERT	Telomerase reverse transcriptase	-1.48	Catalytic subunit of the enzyme telomerase essential for telomerase activity	(56)
MPRIP	Myosin phosphatase Rho-interacting protein	-1.44	Responsible for dephosphorylation of regulatory light chain of myosin, and so negatively regulates actomyosin-based contractility, regulates contractility in vascular smooth muscle	(57)
IGHE	Ig epsilon chain C region	-1.43	IgE associated with allergic responses	
DNAJB6	DnaJ homolog subfamily B member 6	-1.15	Mitochondrial heat shock protein 40, reduces DCM	(58, 59)
FCGR3B	Low affinity immunoglobulin gamma Fc region receptor III-B	-1.00	FcgIII- low affinity receptor for the IgG, associated with immune complex formation in autoimmune diseases	
SPSB4	SPRY domain-containing SOCS box protein 4	-0.80	MHC class I antigen processing	
TAGLN3	Transgelin-3	-0.78	TGFb-inducible protein that regulates stem cells via actin cytoskeleton	(60)
CRP	C-reactive protein	-0.74	Systemic biomarker of inflammation in CVD	(61, 62)
SHBG	Sex hormone-binding globulin	-0.72	Carries estrogen, dihydrotestosterone, and testosterone in the blood of females and males	
SRGN	Serglycin	-0.72	Proteoglycan expressed on all immune cells associated with granules and inflammation	(63)
QPCT	Glutaminyl-peptide cyclotransferase	-0.68	Enzyme involved in post-translational modifications, associated with inflammation	(64)
CIRBP	Cold-inducible RNA binding protein	-0.68	Induced in response to cellular stress, DAMP that activates TLR4	(65)
AGRN	Agrin	-0.67	Induces the aggregation of nicotinic acetylcholine receptors for nerves and neuromuscular junction, elevated levels biomarker for muscle weakness	(66)
MBL2	Mannose-binding protein C	-0.64	MBL recognizes carbohydrate patterns found on the surface of pathogens including bacteria, viruses, protozoa and fungi. Activates complement and lectin pathways.	
LBP	LPS-binding protein	-0.63	Soluble acute-phase protein that binds to bacterial LPS to activate CD14 and TLR4	
HLA-A	HLA class I histocompatibility antigen, A-29 alpha chain	-0.61	HLA class I activation	
NDUFB6	NADH dehydrogenase [ubiquinone] 1 beta subcomplex subunit 6	-0.61	Mitochondrial complex I, inner mitochondrial membrane component	
CD5L	CD5 receptor-like	-0.59	Macrophage anti-inflammatory protein, reduces mast cell and NLRP3 inflammasome activity	(67)

*^a^* AGRN, Agrin; AP-1, activator protein 1; CD5L, CD5 receptor-like; CIRBP, cold-inducible RNA binding protein; CRP, C-reactive protein; CVD, cardiovascular disease; DAMP, damage-associated molecular pattern; DCM, dilated cardiomyopathy; DNAJB6, DNAJ homolog subfamily B member 6; FAM91A1, protein FAM91A1; FCGR3B, low affinity immunoglobulin gamma Fc region receptor III-B; KEL, Kell blood group glycoprotein; HAS1, Hyaluronan synthase 1; HLA, human leukocyte antigen; HLA-A, HLA class I histocompatibility antigen, A-29 alpha chain; Ig, immunoglobulin; IGHE, Ig epsilon chain C region; LBP, lipopolysaccharide binding protein; MBL, mannose-binding lectin; MBL2, mannose-binding protein C; MHC, major histocompatibility complex; MPRIP, myosin phosphatase Rho-interacting protein; NDUFB6, NADH dehydrogenase [ubiquinone] 1 beta subcomplex subunit 6; NLRP3, nucleotide-binding domain, leucine-rich–containing family, pyrin domain–containing-3; QPCT, glutaminyl-peptide cyclotransferase; SHBG, sex hormone-binding globulin; SPSB4, SPRY domain-containing SOCS box protein 4; SRGN, Serglycin; TAGLN3, Transgelin-3; TERT, telomerase reverse transcriptase; TLR4, Toll-like receptor 4; TGFβ, tissue growth factor beta.

**Table 2:** Roles of differentially expressed proteins in pmPEV according to the literature.

Protein	Protein definition	Log2fold change	Roles in literature	References
PLGLB1*^a^*	Plasminogen-like protein B	3.92	Same gene as PLGLB2	
TRAP1	Heat shock protein 75 kDa, mitochondrial	2.01	Protects mitochondria and inhibits inflammation	(68)
PPP1R14A	Protein phosphatase 1 regulatory subunit 14A	1.34	Associated with inflammation in many cancers	(69)
LPA	Apolipoprotein(a)	1.20	Component of HDL, contaminant in EVs	
SAA1	Serum amyloid A-1 protein	1.11	Apolipoprotein associated with HDL, acute phase protein levels go up with infection, tissue injury and cancer, increases inflammation	
CKM	Creatine kinase M	0.96	Serum CK-MB biomarker of cardiac damage in CVDs including myocarditis	(70)
TTR	Transthyretin	0.83	Plasma transport protein for thyroid hormone thyroxine and retinol	
MBP	Myelin basic protein	0.83	Key protein in the myelin sheath of nerves	
S100A6	Protein S100-A6	0.77	Calcium-dependent S100A6 governs AKT activation pathway including regulation of mitochondrial calcium levels, respiratory metabolism, Hsp90 protein, and stem cell survival	(71)
APOC2	Apolipoprotein C2	0.66	Component of LDL and chylomicrons, contaminant of EVs	(72)
TTN	Titin	0.66	Mutations in titin lead to DCM	(73)

*^a^* AKT, protein kinase B; APOC2, Apolipoprotein C2; CKM, creatine kinase M; CK-MB, creatine kinase-myocardial band; CVD, cardiovascular disease; DCM, dilated cardiomyopathy; EVs, extracellular vesicles; kDa, kilodalton; LPA, Apolipoprotein(a); HDL, high density lipoprotein; Hsp90, heat shock protein 90; LDL, low density lipoprotein; MBP, myelin basic protein; PLGLB1, plasminogen-like protein B; PPP1R14A, protein phosphatase 1 regulatory subunit 14A; S100A6, protein S100-A6/calcyclin; SAA1, serum amyloid A-1 protein; TRAP1, heat shock protein 75 kDa; TTN, Titin; TTR, Transthyretin.

### 3.6 PEV and pmPEV share similar proteome to other reported platelet/plasma EVs

**Figure 6:**
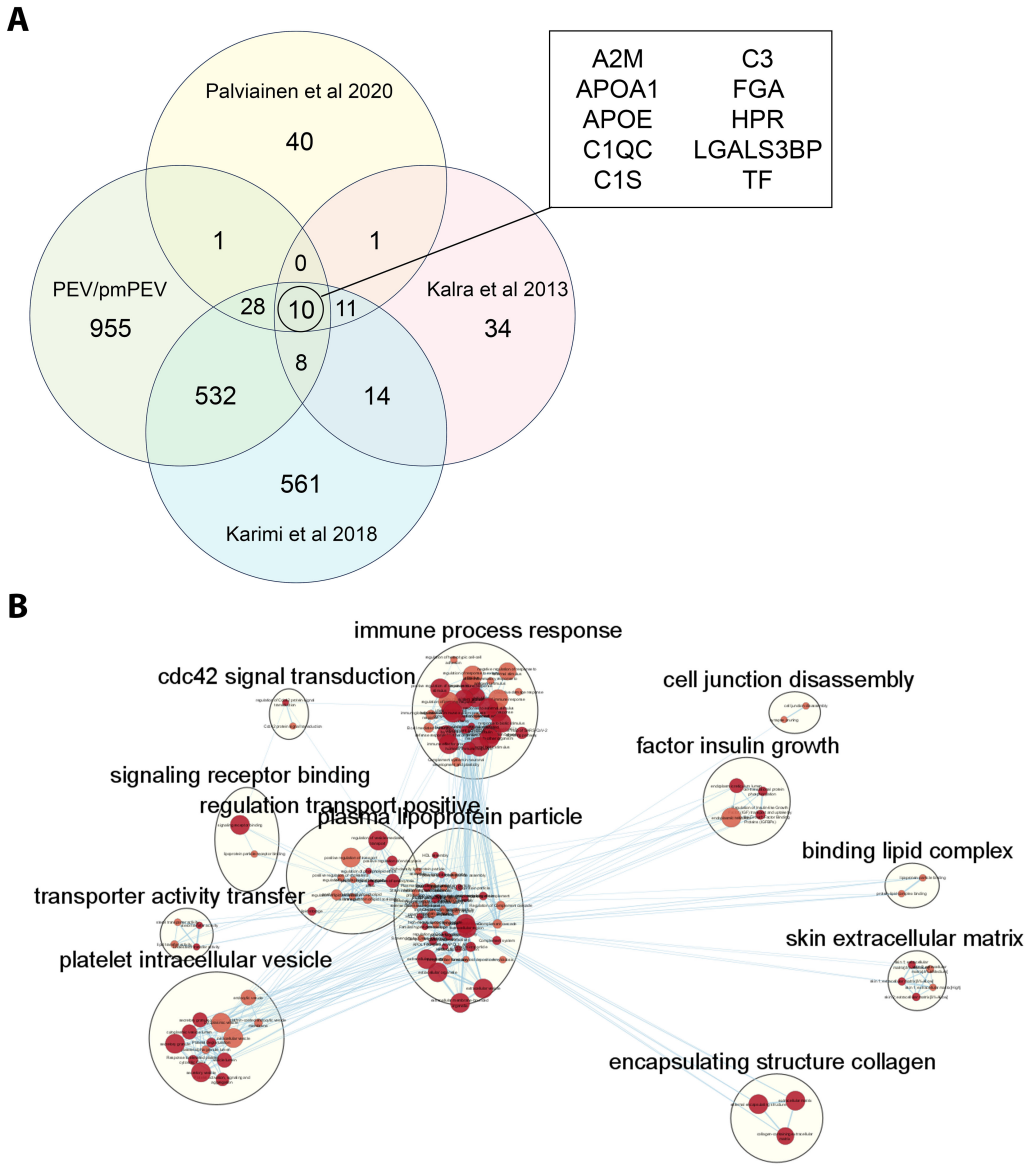
PEV and pmPEV share a proteome with other platelet/plasma EV products. **(A)** 4-way Venn diagram shows shared proteome of PEV and pmPEV compared to 3 other platelet EV products reported in the literature. **(B)** Cytoscape pathway visualization depicts super-clusters of pathways from the shared proteome of all five of these platelet/plasma EV products. The 12 superclusters visualized include: cdc42 signal transduction, immune process response, cell junction disassembly, factor insulin growth, signalling receptor binding, regulation transport positive, plasma lipoprotein particle, binding lipid complex, transporter activity transfer, platelet intracellular vesicle, skin extracellular matrix, and encapsulating structure collagen.

Because we found significant differences in the proteomes of PEV versus pmPEV, we wanted to examine whether our platelet-derived EV products were similar or different from other published proteomes of platelet/plasma EV products isolated using a similar extraction method (i.e., ultracentrifugation). Using a comparison method presented by Palviainen et al., we compared the shared proteome of PEV and pmPEV to the shared proteome of 3 other platelet/plasma EV products from the literature (37–39). A comparison of all shared or distinct proteins between the studies are depicted in a modified 4-way Venn diagram in **Figure 6A**. All 5 products shared 10 proteins including complement components C3, C1qc and C1s as well as the contaminants ApoA1 and ApoE. Other proteins shared between the 5 products included α2-macroglobulin (A2M) [anti-protease in blood, inhibits coagulation, stimulates immune response, and acts as carrier by binding many cytokines in the blood such as IL-1β and TGFβ (77)], fibrinogen A (FGA) (coagulation protein), histidine-containing phosphocarrier protein (HPr) (a small cytoplasmic protein that is a component of the phosphoenolpyruvate-dependent sugar phosphotransferase system/PTS), galectin-3 binding protein (LGALS3BP) [increased after viral infection leading to increased interferons/IFNs (78)], and transferrin (TF) (protein that binds iron in blood serum). We illustrate the shared protein pathway superclusters of these 10 proteins in **Figure 6B** using Cytoscape. Overall, these findings indicate that the shared proteome of PEV and pmPEV is similar to other platelet or plasma-derived products isolated using ultracentrifugation, and that complement components and contamination with apolipoproteins may be a common characteristic of these products. Importantly, both PEV and pmPEV significantly reduced myocarditis in our translational animal model despite this potentially proinflammatory protein content.

### 3.7 miR sequencing reveals enriched anti-inflammatory miR content in PEV and pmPEV, but some proinflammatory content in pmPEV

**Figure 7:**
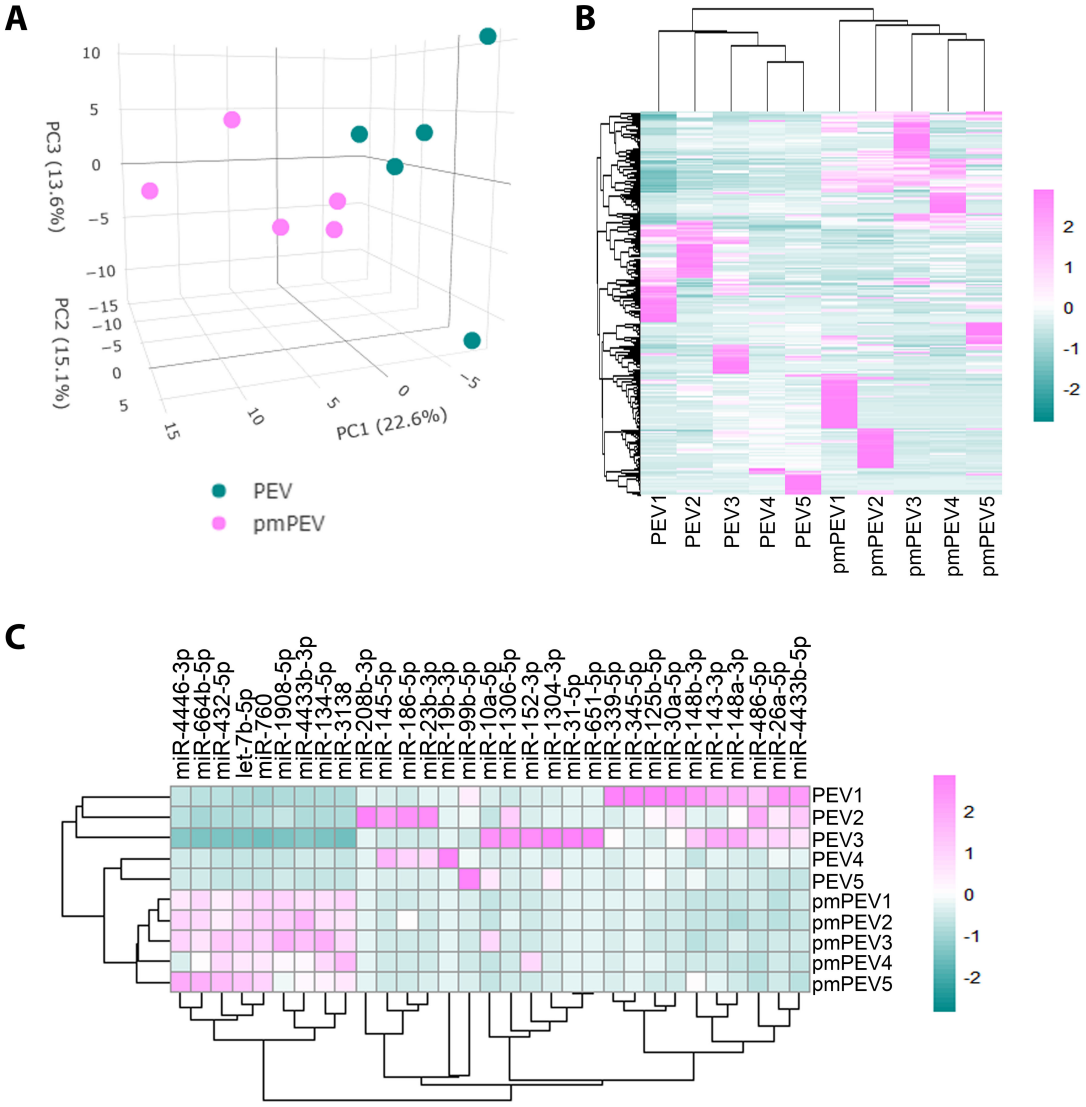
PEV and pmPEV enriched for anti-inflammatory miRs, but pmPEV had a more proinflammatory signature. **(A)** Principal component analysis. **(B)** Hierarchical clustering of miRs. **(C)** Hierarchal clustering of differentially expressed miRs.

We also conducted miRNA (miR) sequencing on PEV and pmPEV products to better understand the effect of miR content on their ability to inhibit myocarditis (**Figure 7**). PCA (**Figure 7A**) and heatmap (**Figure 7B**) showed that PEV and pmPEV samples were different than each other and were similar, but with some variation, within their own groups. We identified 31 miRs that were differentially expressed between PEV and pmPEV (**Figure 7C**).

**Figure 8:**
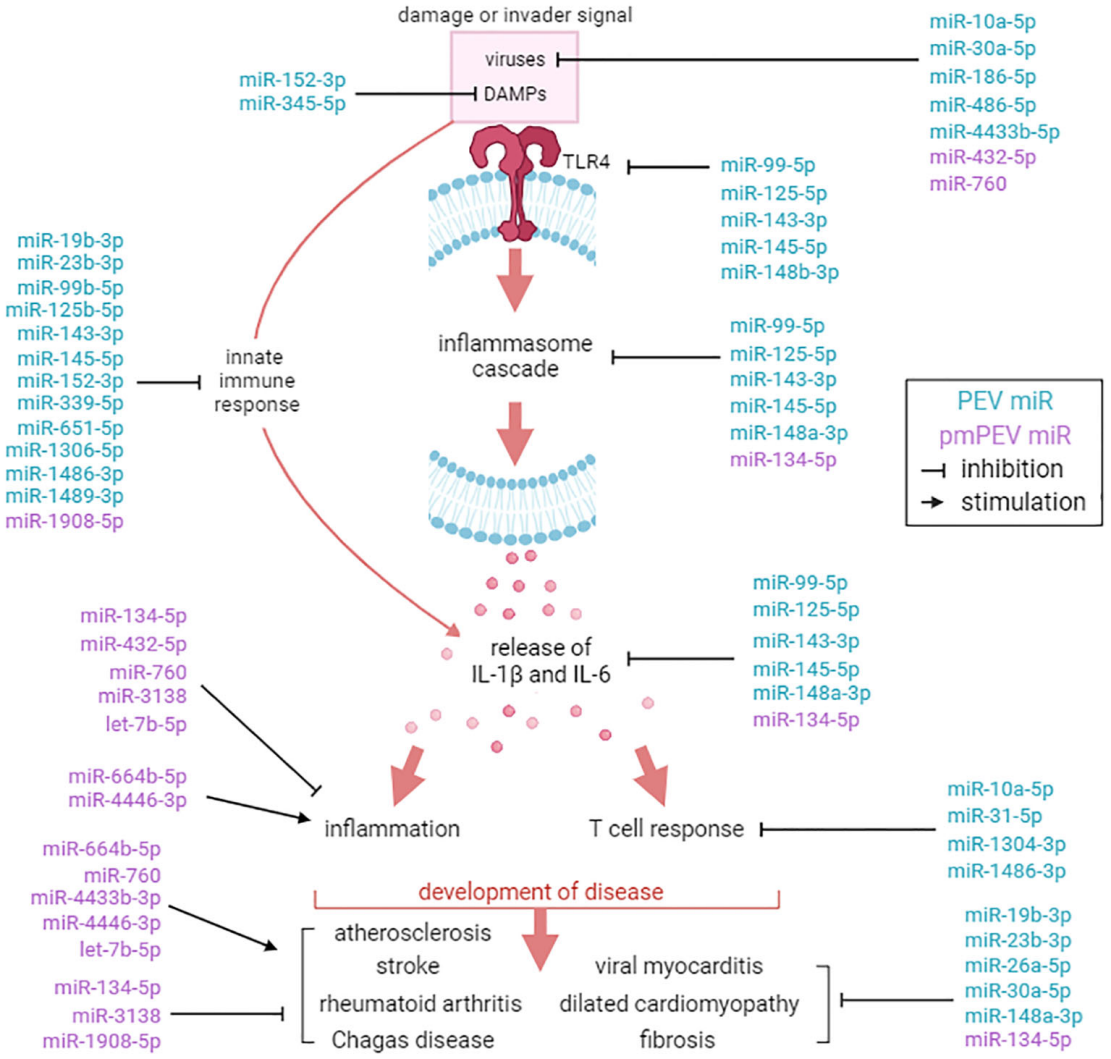
Comparison of miR targets between PEV and pmPEV. Mechanistic effects of miRs reported in the literature identified in PEV (blue) versus pmPEV (purple) that influence key pathways in the pathogenesis of viral myocarditis. References and descriptions of miRs found in **Tables 3**, **4**. This figure was created using BioRender.com.

A literature review was conducted for the 31 miRs to better understand how they could modulate the primary gene pathways. The findings from the literature search are shown in **Tables 3**, **4** and illustrated in **Figure 8**. Both PEV and pmPEV contained miRs that inhibited key pathways of the pathogenesis of myocarditis like the TLR4/inflammasome pathway. Remarkably, all the miRs in PEV were reported in the literature to be anti-viral, anti-inflammatory, to inhibit the TLR4/inflammasome pathway, or had been shown to decrease CVB3 myocarditis in an animal model (80, 85–87) (**Table 3**), which correlates to the global shut-down of myocardial inflammation observed for PEV. In contrast, pmPEV had three miRs that were proinflammatory out of 9 distinct miRs (33%) with the remaining 6 having generally inhibitory roles according to the literature (**Table 4**). Overall, the miR content of PEV and pmPEV were consistent with the histologic, qRT-PCR, and IHC data showing that PEV more effectively regulated the immune response during myocarditis than pmPEV.

**Table 3:** Published targets for top miRs enriched in PEV*^a^*.

Human miR	Log2 fold change	Published role	Reference
miR-10a-5p	3.712	Inhibits viral infections & T cells	(79)
miR-19b-3p	5.021	Protects against CVB3 myocarditis by decreasing inflammation and switching to protective M2 macrophages	(80)
miR-23b-3p	5.196	Reduces cardiomyopathy by targeting/reducing MyD88-induced NFkB	(81, 82)
miR-26a-5p	6.219	Reduces fibrosis in lung, reduced in DCM patients and protects heart in animal model	(83, 84)
miR-30a-5p	6.219	Increased with CVB3 infection, protects against CVB3 myocarditis	(85–87)
miR-31-5p	4.347	Targets CD40L and SAP to prevent activation of T helper cells	(88)
miR-99b-5p	4.326	Inhibits NLRP3 inflammasome, inhibits PI3K/AKT/mTOR signaling	(89, 90)
miR-125b-5p	4.944	Associated with a number of cardiovascular diseases, but shown to inhibit IL-1b by targeting TRAF6/MAPK/NFkB signaling	(91–94)
miR-143-3p	1.967	Decreases TLR4, MyD88, and NFkB and increases IL-10	(95)
miR-145-5p	5.909	Decreases TLR4 and PI3K/AKT/mTOR signaling	(96, 97)
miR-148a-3p	1.727	Inhibits IL-1b damaging effects and is inhibited by IL-1b, inhibits NF-κB during acute viral myocarditis	(98, 99)
miR-148b-3p	3.375	Increases proliferation of MSCs and Th2 responses that inhibit TLR4	(100, 101)
miR-152-3p	4.154	Decreases PI3K/AKT signaling, inhibits mitochondrial autophagy	(102, 103)
miR-186-5p	4.096	Increases anti-viral beta-defensin-1	(104)
miR-208b-3p	3.779	Biomarker of sudden cardiac death, but fibroblasts treated with this miR when implanted in myocardial infarcts regenerated the heart and improved cardiac function	(105, 106)
miR-339-5p	6.945	Targets NEAT1 which regulates innate immune function	(107, 108)
miR-345-5p	5.458	Targets Drp1 and induces mitochondrial fission, decreases fibrosis by targeting SARS	(84, 109)
miR-486-5p	2.120	Increased with viral infections, but anti-viral role	(110, 111)
miR-651-5p	4.716	Targets TM4SF4, CD34 (cell-to-cell adhesion), Cxcl10 (M1 chemokine), STMN1 (cell proliferation and migration), LAMNC1 (laminin- cell migration, etc.), and others…	(112)
miR-1304-3p	5.794	Targets GATA2, which is important for normal immune function	(113)
miR-1306-5p	4.398	Targets SLCO2A1 to inhibit PI3K/AKT/mTOR signaling	(114)
miR-4433b-5p	2.475	Low serum levels predict mortality in COVID patients	(115)

*^a^* AKT, protein kinase 3; CD34, cluster of differentiation 34; CD40L, cluster of differentiation 40 ligand; COVID, Coronavirus Disease of 2019; CVB3, Coxsackievirus B3; Cxcl10, C-X-C motif chemokine ligand 10; DCM, dilated cardiomyopathy; Drp1, Dynamin-related protein 1; GATA2, GATA-binding factor 2; IL-1β, interleukin 1 beta; IL-10, interleukin 10; LAMNC1, Laminin Gamma 1 Chain; MAPK, Mitogen-activated protein kinase; mIR, micro RNA; MSCs, mesenchymal stem cells; mTOR, mammalian target of rapamycin; MyD88, MYD88 Innate Immune Signal Transduction Adaptor; NFκB, nuclear factor kappa B; NEAT1, Nuclear Paraspeckle Assembly Transcript 1; NLRP3, NOD- LRR- and pyrin domain-containing protein 3; PI3K, phosphatidylinositol 3-kinase; SAP, SLAM-associated protein; SLCO2A1, Solute Carrier Organic Anion Transporter Family Member 2A1; STMN1, stathmin 1; Th2, T helper 2; TLR4, Toll-like receptor 4; TMASF4, transmembrane 4 L six family member 4; TRAF6, tumor necrosis factor receptor associated factor 6.

**Table 4:** Published targets for top miRs enriched in pmPEV.

Human miR*^a^*	Log2 fold change	Published role*^b^*	Reference
Let-7b-5p	1.551	Inhibits inflammation, targets mitochondrial cytochrome b reducing reactive oxygen species in cardiomyocytes	(116–118)
miR-134-5p	1.598	Inhibited STAT3/IL-6, improved atherosclerosis	(119, 120)
miR-432-5p	1.604	Anti-viral and anti-inflammatory	(121, 122)
**miR-664b-5p**	1.645	Increases AKT signaling to increase heart disease and remodeling	(123)
miR-760	1.658	Anti-viral and anti-inflammatory	(124–126)
miR-1908-5p	1.661	Improved mitochondrial function inhibiting mTOR and AKT; inhibits rheumatoid arthritis	(127, 128)
miR-3138	1.717	Activates NRF2/AMPK to improve mitochondrial function and reduce heart disease in Chagas	(129, 130)
**miR-44332-3p**	1.519	Risk factor for stroke; associated with morbid obesity and type II diabetes	(131, 132)
**miR-4446-3p**	1.688	Predictor for atherosclerosis in patients with rheumatoid arthritis	(133)

*^a^* Bold miRs in pmPEV associated with increasing inflammation based on current literature.

*^b^* AKT, protein kinase 3; AMPK, adenosine monophosphate-activated protein kinase; mIR, micro RNA; IL, interleukin; mTOR, mammalian target of rapamycin; NRF2, nuclear factor erythroid 2-related factor 2; STAT3, signal transducer and activator of transcription 3.

## 4 Discussion

EVs obtained from patients are typically extracted from healthy volunteers, yet little specific information about the donors is obtained/reported. In this study, we aimed to better understand the effect of donor sex and age on the effectiveness of EVs as a therapy for viral myocarditis. We hypothesized that EVs derived from platelets from healthy female volunteers under the age of 50, which we termed premenopausal PEV or pmPEV, would be more effective at inhibiting myocarditis than PEV obtained from healthy male and female volunteers of all ages. However, we found that PEV was more effective than pmPEV at inhibiting myocarditis, although both products were anti-inflammatory and significantly reduced myocarditis. Additionally, miR content most closely aligned with the effectiveness of each product to reduce myocarditis. All of the miRs enriched in PEV had reported roles in the literature that inhibited specific pathways known to be involved in the pathogenesis of viral myocarditis including reducing viral replication, TLR4 signaling, inflammasome/NLRP3, and IL-1β (**Table 3**). Incredibly, several of the miRs enriched in PEV have already been tested in CVB3 infection, CVB3 myocarditis models, and/or cardiomyopathy/DCM models, where they were found to decrease disease (81, 83–85, 87, 98). In contrast, although most miRs in pmPEV were anti-inflammatory, three miRs were reported to have proinflammatory effects (**Table 4**). Additionally, the anti-inflammatory miRs enriched in pmPEV were less specific for pathways known to drive myocarditis and were generally anti-inflammatory (**Table 4**). Importantly, even though PEV and pmPEV had distinct proteomes and transcriptomes, both products were able to significantly decrease myocarditis. This finding indicates that even though batches of EV products obtained from different healthy donors differ in proteomic and miR content, their overall potential for therapeutic effectiveness is high. Additionally, by examining proteomics, we showed that the miR profile of these products was more important than their proteomic profile at determining the effectiveness of treatment.

Our data also suggest that regenerative therapy strategies that pool miRs that specifically and simultaneously target multiple stages of the known pathogenesis of disease may be highly effective at preventing inflammation. In this study we identified 31 miRs that very effectively reduced viral myocarditis in a highly translational animal model. Importantly, many of these miRs should also reduce remodeling, and thus have the potential to reduce progression to chronic inflammatory cardiomyopathy/DCM in addition to decreasing acute myocarditis. We did not observe miRs that have been reported in the literature to inhibit mast cell activation/degranulation in either product. If the products had also contained this miR content, they may have more effectively reduced pericarditis/perimyocarditis. Additionally, the proteomic content of both of the tested products contained several factors including IgE and IgG and/or their components that could have activated mast cells, thereby counteracting the anti-inflammatory actions of the miR content. We also did not observe other miRs that may reduce myocarditis such as miR-122, which has been found to inhibit tripartite motif-containing protein 29 (TRIM29) where low levels of TRIM29 reduce viral replication and inflammation in a fulminant model of viral myocarditis (134–136).

Myocarditis patients lack disease-specific therapies due to the broad innate and adaptive immune mechanisms involved in the pathogenesis of disease, and so this disease presents an ideal model for testing a heterogeneous product that may be able to globally reduce a number of specific pathways- an approach that specific targeted therapy cannot accomplish. EV-based products offer promising potential as ‘global’ therapies, as they carry a variety of functional DNA, RNA, miR, and protein cargo that can provide the broad, yet targeted, messaging needed to effectively control viral infection, inflammation and remodeling that drives the pathogenesis of myocarditis.

## 5 Limitations

It remains unclear whether the age and sex of the donor products affected the miR and protein content of the EVs. One possibility is that higher estrogen levels in younger women under age 50 resulted in a more robust proinflammatory profile as estrogen at high levels/doses is known to exert proinflammatory effects but at low levels/doses to be anti-inflammatory (137, 138). Another possibility is that men develop more robust immune responses to CVB infection, as we observe when we examine sex differences in viral myocarditis (14), that generates more protective miRs in their EVs. CVB3 is a common infection worldwide with individuals in the population being reinfected with different serotypes of enteroviruses each year, similar to colds and the flu (139), and so it is not surprising to find miRs that target CVB in our donor EVs. A different experimental approach is needed in the future to identify the effect of sex and age donor parameters on the EV protein and miR content, likely using individually based products instead of pooling, so that specific donor contributions can be identified, or testing several independent batches of products with donor pools that match more specifically according to sex and age. Additionally, we injected the EVs during the innate immune response to viral infection, which is the optimal time to test the effect of the product on myocarditis, as we showed previously (12, 33); however, this is not a clinically relevant timepoint. We showed previously that PEV given during acute myocarditis at days 7, 8, and 9 pi was able to significantly reduce myocarditis and improve cardiac function (12), suggesting these products can also be used effectively at clinically relevant time points. Future studies should also examine the effect of PEV/pmPEV and/or miRs given intravenously or by intracardiac injection, which are more clinically relevant routes of administration. While EVs are known to contain the functional contents that we tested, this study could not confirm the source of the proteins and miRs identified from these bulk, heterogenous products. For example, the miRs we identified could be present *within* the EVs in PEV and pmPEV, associated with the external EV protein corona as has been shown elsewhere (140), or present in the bulk products as extracellular miRs, which are stable in plasma (141). The focus of the study was to primarily characterize the differences of the products; a clearer understanding of the roles played by components in immunoregulation during myocarditis would require functional testing of candidate proteins and the miRs we identified. Research has shown common contaminants, including some lipoproteins like high density lipoprotein, can also have anti-inflammatory and cardioprotective effects (142), and it is not clear from this work to what extent those lipoproteins participated in the anti-inflammatory effects we observed by PEV and pmPEV. Future research should determine the ability of each miR to effectively inhibit viral myocarditis during a clinically relevant timepoint, although likely the aggregate effect of these miRs heavily contributed to the broad reduction of myocarditis without immunosuppression during viral infection.

## 6 Conclusions

We found that PEV and pmPEV products significantly decreased myocardial inflammation in a highly translational mouse model of viral myocarditis. We identified 31 miRs that specifically targeted key pathways in the pathogenesis of myocarditis according to the literature that are candidates for future miR-related therapies for viral myocarditis.

## Data Availability

The miRNA sequencing data presented in the study are deposited in the NCBI GEO repository, accession number GSE283563. The proteomic data are deposited in the PRIDE Proteomics repository, accession number PXD058698.
