# Supplementary material for: Therapeutic effects of platelet-derived extracellular vesicles on viral myocarditis correlate with biomolecular content
Source: Front Immunol. 2025 Jan 6;15:1468969. doi: 10.3389/fimmu.2024.1468969 (PMC11743460; doi:10.3389/fimmu.2024.1468969)
Supplement: Supplementary file 1 [file DataSheet1.docx]

**Supplementary Data**

**Therapeutic effects of platelet-derived extracellular vesicles on viral myocarditis correlate with biomolecular content**

**Danielle J. Beetler^1-3^, Presley Giresi^1^, Damian N. Di Florio^1-3^, Jessica J. Fliess^1^, Elizabeth J. McCabe^1^, Molly M. Watkins^1-3^, Vivian Xu^1^, Matthew E. Auda^1^, Katelyn A. Bruno^1,4^, Emily R. Whelan^1-3^, Stephen P.C. Kocsis^1^, Brandy H. Edenfield^5^, Sierra A. Walker^3,6,7^, Logan P. Macomb^1^, Kevin C. Keegan^1^, Angita Jain^1-3^, Andrea C. Morales-Lara^1^, Isha Chekuri^1^, Anneliese R. Hill^1^, Houssam Farres^8^, Joy Wolfram^9,10^, Atta Behfar^7,11^, Paul G. Stalboerger^11^, Andre Terzic^11,12^, Leslie T. Cooper, Jr.^1^^, DeLisa Fairweather^1,2,13^*^**

^1^Department of Cardiovascular Medicine, Mayo Clinic, Jacksonville, Florida 32224 USA

^2^Center for Clinical and Translational Science, Mayo Clinic, Rochester, Minnesota 55902 USA

^3^Mayo Clinic Graduate School of Biomedical Sciences, Mayo Clinic, Rochester, Minnesota 55902 USA

^4^Division of Cardiovascular Medicine, University of Florida, Gainesville, Florida 32608 USA

^5^Department of Cancer Biology, Mayo Clinic, Jacksonville, Florida 32224 USA

^6^Center for Systems Biology, Massachusetts General Hospital, Boston, Massachusetts 02114 USA

^7^Department of Molecular Pharmacology & Experimental Therapeutics, Mayo Clinic, Rochester, Minnesota 55905 USA

^8^Department of Vascular Surgery, Mayo Clinic, Jacksonville, Florida 32224 USA

^9^School of Chemical Engineering, The University of Queensland, Brisbane, Queensland 4072 Australia

^1^Australian Institute for Bioengineering and Nanotechnology, The University of Queensland, Brisbane, Queensland 4072 Australia

^11^Van Cleve Cardiac Regenerative Medicine Program, Mayo Clinic Center for Regenerative Medicine, Rochester, Minnesota 55902 USA

^12^ Department of Clinical Genomics, Mayo Clinic, Rochester, Minnesota 55905 USA

^13^ Department of Immunology, Mayo Clinic, Jacksonville, Florida 32224 USA

^Co-senior authors

***Correspondence:**DeLisa Fairweather, PhD

[Fairweather.DeLisa@mayo.edu](mailto:Fairweather.DeLisa@mayo.edu)

**Supplemental Figures**

**
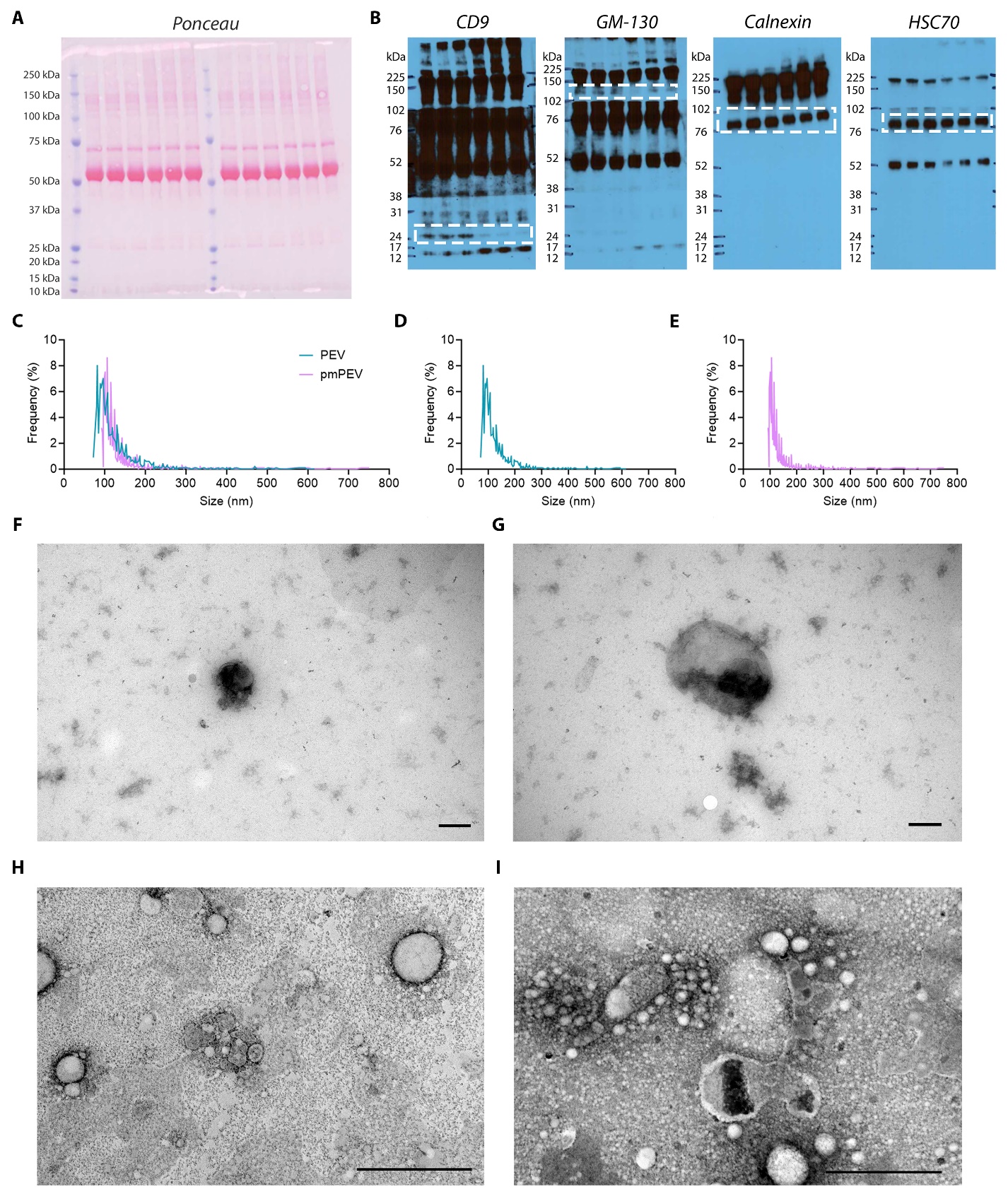
Supplementary Figure 1.** (A) Ponceau stain of western blot. (B) Full images of western blots depicted in Figure 1C. (C) Combined Exoid size distribution spectrum of PEV (teal) and pmPEV (purple). Individual size distributions are shown for (D) PEV and (E) pmPEV. Widefield transmission electron microscopy images show visualization of (F, H) PEV and (G, I) pmPEV samples. (A) Magnification 60 kx, Scale bars: 200 nm; (B) Magnification 60 kx, Scale bars: 200 nm; (C) Magnification 50 kx, Scale bars: 1000 nm; (D) Magnification 50 kx, Scale bars: 1000 nm.


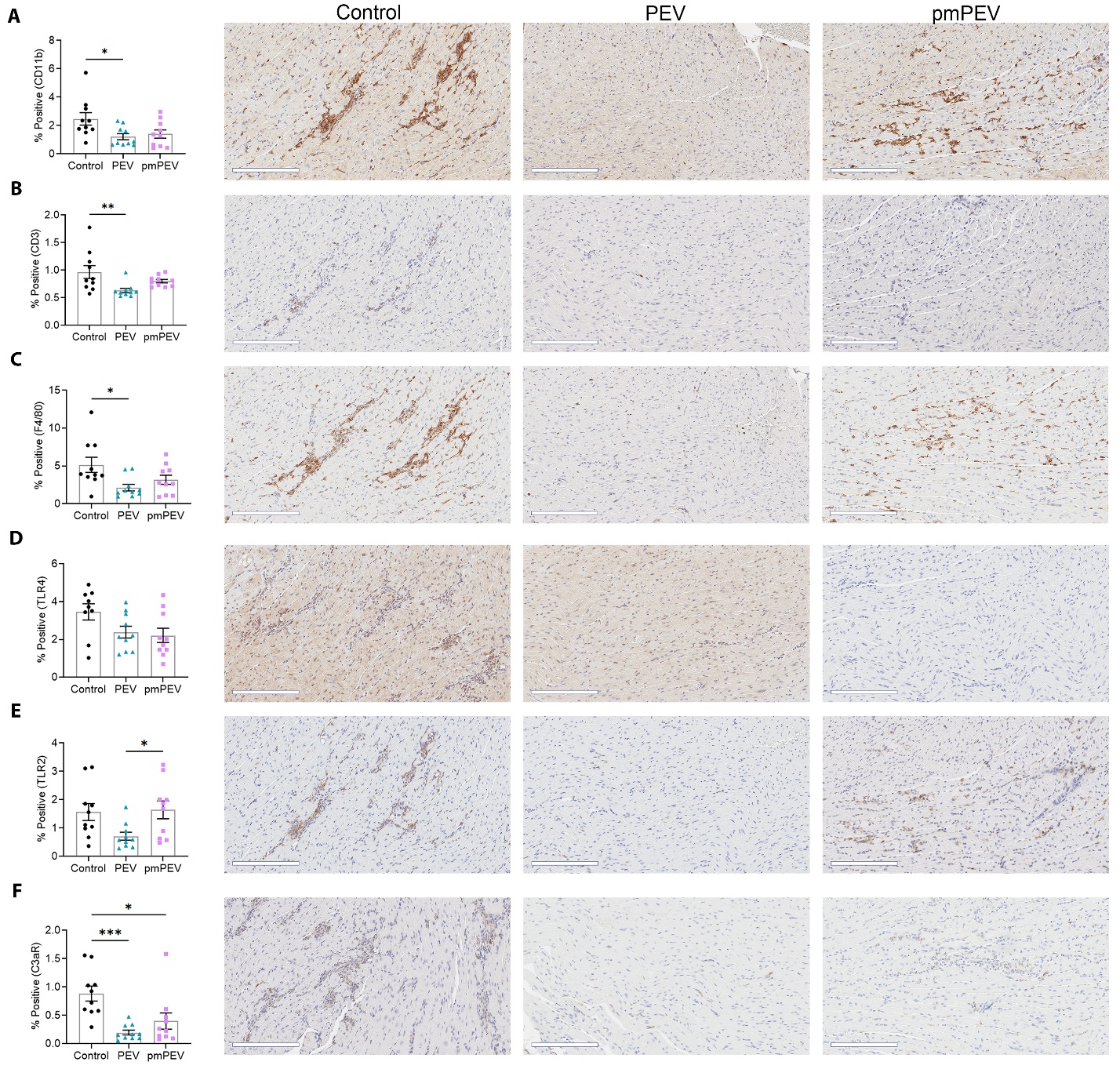
 **Supplementary Figure 2. Immunohistochemistry showed more immune cell reduction by PEV than pmPEV.** EVs from healthy men and women (PEV), or from healthy premenopausal women (pmPEV) (0.25 mL of 10^10^ NPs/mL), or control (0.25 mL of 1X PBS) were given to male BALB/c 8-week-old mice ip on days -1, 0, 1 pi with 10^3^ PFU of CVB3 given ip on day 0. Representative images of immunohistochemical staining with antibodies against A) CD11b, B) CD3, C) F4/80, D) TLR4, E) TLR2, and F) C3aR; *scale bar*: 200 µm. Summary data (*left*) were expressed as positive pixels were compared to total positive and negative pixels to determine % positive. Data show mean ±SEM for 10 mice/group, one-way ANOVA with Dunnett’s multiple comparison; *, *p* < 0.05; **, *p* < 0.01; ***, *p* < 0.001 (see **Supplementary Table 5**).

**
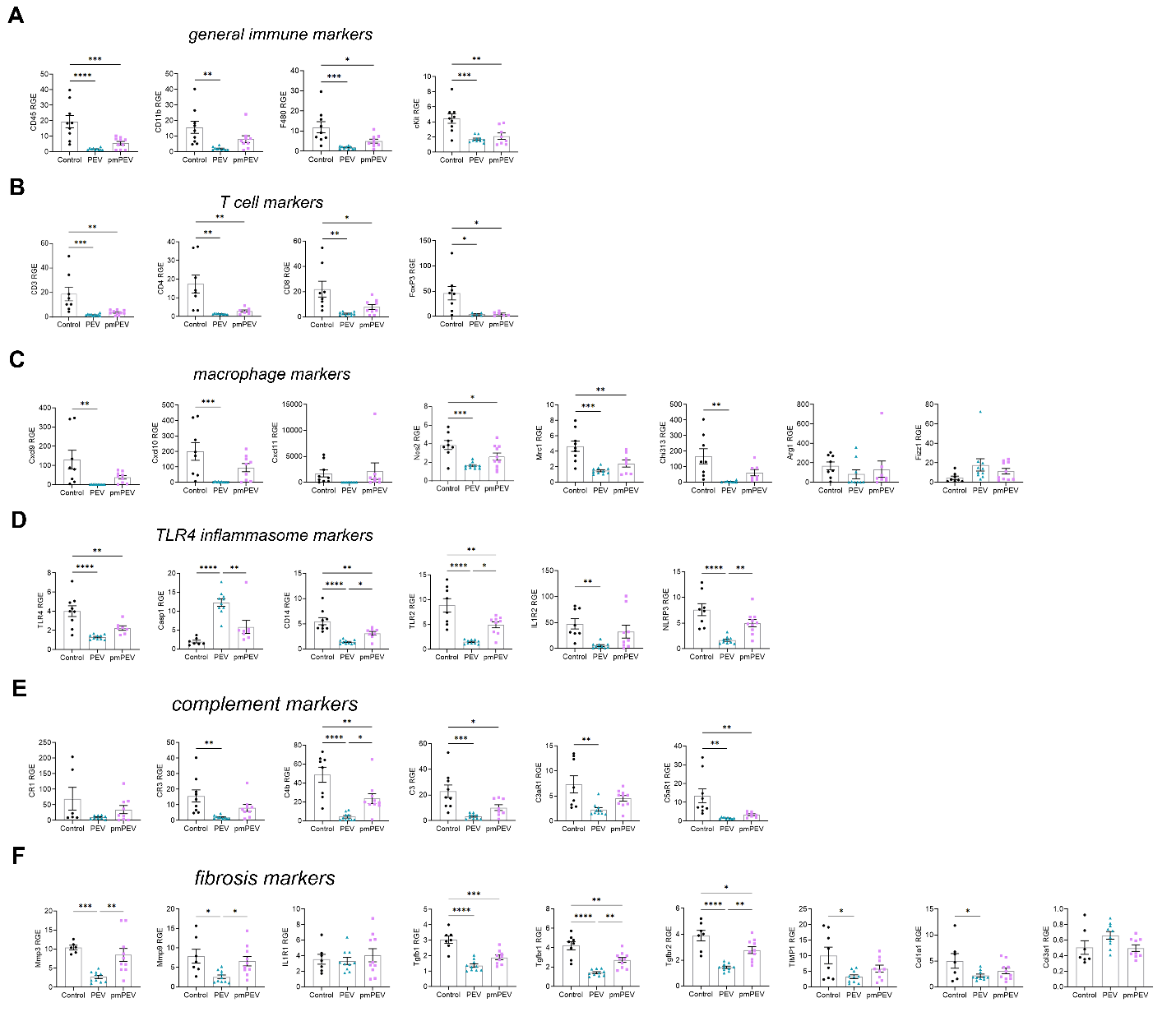
**

**Supplementary Figure 3. Innate treatments with PEV or pmPEV globally decreased immune markers of acute myocarditis and fibrosis pathways.** Nanoparticles (NPs) from healthy men and women (PEV), or from healthy premenopausal women (pmPEV) (0.25 mL of 10^10^ NPs/mL), or Control (0.25 mL of 1X PBS) were given to male BALB/c 8 week (wk) old mice intraperitoneally (ip) on days -1, 0, 1 post infection (pi) with 10^3^ PFU of CVB3 given ip on day 0. Relative gene expression (RGE) by qRT-PCR of day 10 pi hearts was used to assess (A) general immune markers: CD45 (total lymphocytes), CD11b^+^ cells (leukocytes – granulocytes, macrophages, mast cells), F4/80^+^ macrophages, and receptor tyrosine kinase^+^ (*ckit*) (mast cells); (B) T cells: CD3^+^ T cells, CD4^+^ T cells, CD8^+^ T cells, and FoxP3^+^ T regulatory cells; (C) macrophage markers: Chemokine (C-X-C motif) ligand 9 (*Cxcl9*), Chemokine (C-X-C motif) ligand 10 (*Cxcl10*), Chemokine (C-X-C motif) ligand 11 (*Cxcl11*), nitric oxide synthase 2 (*Nos2*), Mannose receptor C-type 1 (*Mrc1*), Chitinase-like 3 (*Chi313*), Arginase 1 (*Arg1*), and found in inflammatory zone 1 (*Fizz1*); (D) TLR4 inflammasome markers: Toll-like receptor 4^+^ (*TLR4*) cells, Caspase 1 (*Casp1*), *CD14*, *TLR2*, Interleukin 1 receptor 2 (*IL1R2*), and nucleotide-binding domain, leucine-rich–containing family, pyrin domain–containing-3 (*Nlrp3*); (E) complement markers: complement receptor 1 (*CR1*), complement receptor 3 (*CR3*), complement component 4b (*C4b*), complement component 3 (*C3*), complement component 3 antagonist receptor 1 (*C3aR1*), and complement component 5 antagonist receptor 1 (*C5aR1*); and (F) fibrosis markers: Matrix metalloproteinase 3 (*Mmp3*), Matrix metalloproteinase 9 (*Mmp9*), interleukin 1 receptor 1 (*IL1R1*), tissue growth factor beta 1 (*Tgfb1*), tissue growth factor beta receptor 1 (*Tgfbr1*), tissue growth factor beta receptor 2 (*Tgfbr2*), Tissue inhibitor of metalloproteinase (*TIMP1*), Collagen Type I Alpha 1 (*Col1a1*), Collagen Type 3 Alpha 1 and (*Col3a1*), all compared to the housekeeping gene, hypoxanthine-guanine phosphoribosyltransferase (*Hprt*). Data show mean ± SEM for 10 mice/group, one-way ANOVA with Dunnett’s multiple comparison. *, *p* < 0.05; **, *p* < 0.01; ***, *p* < 0.001; ****, *p* < 0.0001. Data show mean ± SEM for 10 mice/group, one-way ANOVA with Dunnett’s multiple comparison.

**Supplemental Tables**

**Supplementary Table S1. Western primary antibody solution details**

| **Antibody** | **Dilution** | **1˚ Block** | **Catalog Number** | **Clone** | **Host Species** | **Company** | **Location** |
| --- | --- | --- | --- | --- | --- | --- | --- |
| Calnexin | 1:5000 | 5% BSA in TBST | ab92573 | EPR3632 | rabbit | Abcam | Cambridge, United Kingdom |
| CD9 | 1:1000 | 1% milk in TBST | 13174s | D8O1A | rabbit | Cell Signaling | Danvers, Massachusetts, USA |
| GM-130 | 1:2000 | 5% BSA in TBST | 610822 | 35/GM130 | mouse | BD Biosciences | Franklin Lakes, New Jersey, USA |
| HSC70 | 1:2000 | 5% BSA in TBST | ab51052 | EP1531Y | rabbit | Abcam | Cambridge, United Kingdom |

*Abbreviations*: BSA, Bovine serum albumin; GM-130, Golgi matrix protein 130 kilo Daltons; HSC70, heat shock chaperone protein 70; TBST, Tris-buffered saline with Tween 20; USA, United States of America.

**Supplementary Table S2. qRT-PCR probe sets**

| **Gene** | **Full Name/Marker** | **Therm-Scientific Catalog #** |
| --- | --- | --- |
| *Arg1* | Arginase 1 | Mm00475988_m1 |
| *C3* | complement component 3 | Mm01232779_m1 |
| *C3aR1* | complement component 3 antagonist receptor 1 | Mm02620006_s1 |
| *C4b* | complement component 4b | Mm00437893_g1 |
| *C5aR1* | complement component 5 antagonist receptor 1 | Mm00500292_s1 |
| *Casp1* | Caspase 1 | Mm00438023_m1 |
| *CD11b* | Myeloid lineage immune marker | Mm00434455_m1 |
| *CD14* | Inflammasome marker | Mm00438094_g1 |
| *CD3e* | T cell marker | Mm01179194 |
| *CD4* | Helper T cell marker | Mm00442754_m1 |
| *CD45* | Immune cell marker | Mm00448522_m1 |
| *CD8a* | Cytotoxic T cell marker | Mm01182107_g1 |
| *Chi313* | mouse chitinase 3-like 3 | Mm00657889_m1 |
| *cKit* | tyrosine-protein kinase Kit | Mm00445212_m1 |
| *Col1a1* | Collagen Type I Alpha 1 | Mm00801666_g1 |
| *Col3a* | Collagen Type 3 Alpha 1 | Mm00802300_m1 |
| *CR1* | complement receptor 1 | Mm00801681_m1 |
| *Cxcl10* | C-X-C motif chemokine ligand 10 | Mm00445235_m1 |
| *Cxcl11* | C-X-C motif chemokine ligand 11 | Mm00444662_m1 |
| *Cxcl9* | C-X-C motif chemokine ligand 9 | Mm00434946_m1 |
| *F4/80* | EGF-like module-containing mucin-like hormone receptor-like 1 | Mm00802529_m1 |
| *Fizz1* | found in inflammatory zone 1 | Mm00445109_m1 |
| *FoxP3* | forkhead box P3 | Mm00475162_m1 |
| *HPRT* | hypoxanthine-guanine phosphoribosyltransferase | Mm03024075_m1 |
| *IL1R1* | Interleukin 1 receptor 1 | Mm00516117_m1 |
| *IL1R2* | Interleukin 1 receptor 2 | Mm00439622_m1 |
| *Mmp3* | matrix metalloproteinase 3 | Mm00440295_m1 |
| *Mmp9* | matrix metalloproteinase 9 | Mm00442991_m1 |
| *Mrc1* | mannose receptor C-Type 1 | Mm00485148_m1 |
| *Nlrp3* | NOD- LRR- and pyrin domain-containing protein 3 | Mm00840904_m1 |
| *Nos2* | nitric oxide synthase 2 | Mm00440502_m1 |
| *Tgfb1* | tissue growth factor beta 1 | Mm01178820_m1 |
| *Tgfbr1* | tissue growth factor beta receptor 1 | Mm00436971_m1 |
| *Tgfbr2* | tissue growth factor beta receptor 2 | Mm00436955_m1 |
| *TIMP1* | TIMP metallopeptidase inhibitor 1 | Mm01341361_m1 |
| *TLR2* | Toll like receptor 2 | Mm00442346_m1 |
| *TLR4* | Toll like receptor 4 | Mm00445273_m1 |

**Supplementary Table 3. One-way ANOVA analysis with Dunnet’s multiple comparisons test of H&E and trichrome blue staining**

|  | Control vs. PEV | Control vs. pmPEV | PEV vs. pmPEV | ANOVA value |
| --- | --- | --- | --- | --- |
| myocarditis | 0.0002 | 0.0036 | 0.5189 | 0.0002 |
| pericarditis | 0.6383 | 0.7779 | 0.9707 | 0.4479 |
| fibrosis | 0.0079 | 0.0601 | 0.6630 | 0.008 |

*Abbreviations*: ANOVA, analysis of variance; PEV, platelet-derived extracellular vesicles; pmPEV, pre-menopausal platelet derived extracellular vesicles.

**Supplementary Table 4. One-way ANOVA analysis with Dunnet’s multiple comparisons test of IHC staining**

|  | Control vs. PEV | Control vs. pmPEV | PEV vs. pmPEV | ANOVA value |
| --- | --- | --- | --- | --- |
| CD45 | 0.0187 | 0.0725 | 0.8139 | 0.0174 |
| CD11b | 0.0305 | 0.0713 | 0.9186 | 0.0245 |
| CD3 | 0.0087 | 0.2628 | 0.2485 | 0.0120 |
| F4/80 | 0.0177 | 0.1489 | 0.5788 | 0.0211 |
| TLR4 | 0.1310 | 0.0691 | 0.9403 | 0.0603 |
| TLR2 | 0.0759 | 0.9763 | 0.0489 | 0.0348 |
| C3aR | 0.0007 | 0.0157 | 0.4408 | 0.0008 |

*Abbreviations*: ANOVA, analysis of variance; C3aR, complement receptor 1 antagonist; CD, cluster of differentiation; F4/80, EGF-like module-containing mucin-like hormone receptor-like 1; PEV, platelet-derived extracellular vesicles; pmPEV, pre-menopausal platelet derived extracellular vesicles; TLR, Toll-like receptor.

**Supplementary Table 5. One-way ANOVA analysis with Dunnet’s multiple comparisons test for individual markers from the gene matrices**

|  | ThermoFisher catalog # | Control vs. PEV | Control vs. pmPEV | PEV vs. pmPEV | ANOVA value |
| --- | --- | --- | --- | --- | --- |
| *CVB3* | n/a | 0.1227 | 0.7367 | 0.4141 | 0.1392 |
| *CD45* | Mm00448522_m1 | <0.0001 | 0.0006 | 0.4434 | <0.0001 |
| *CD11b* | Mm00434455_m1 | 0.0018 | 0.1046 | 0.2171 | 0.0026 |
| *F4/80* | Mm00802529_m1 | 0.0005 | 0.0184 | 0.3521 | 0.0006 |
| *cKit* | Mm00445212_m1 | 0.0003 | 0.0031 | 0.7866 | 0.0003 |
| *CD3* | Mm01179194 | 0.0007 | 0.0022 | 0.8746 | 0.0005 |
| *CD4* | Mm00442754_m1 | 0.001 | 0.0047 | 0.9151 | 0.0007 |
| *CD8* | Mm01182107_g1 | 0.0012 | 0.0201 | 0.5076 | 0.0015 |
| *FoxP3* | Mm00475162_m1 | 0.0265 | 0.0213 | 0.9988 | 0.0100 |
| *Cxcl9* | Mm00434946_m1 | 0.0047 | 0.0535 | 0.5594 | 0.0057 |
| *Cxcl10* | Mm00445235_m1 | 0.0009 | 0.0990 | 0.1266 | 0.0014 |
| *Cxcl11* | Mm00444662_m1 | 0.3641 | 0.9388 | 0.2293 | 0.2104 |
| *Nos2* | Mm00440502_m1 | 0.0004 | 0.0486 | 0.1174 | 0.0007 |
| *Fizz1* | Mm00445109_m1 | 0.1167 | 0.5560 | 0.5309 | 0.1375 |
| *Chi313* | Mm00657889_m1 | 0.0019 | 0.0618 | 0.377 | 0.0025 |
| *Mrc1* | Mm00485148_m1 | 0.0001 | 0.0073 | 0.3287 | 0.0001 |
| *Arg1* | Mm00475988_m1 | 0.5655 | 0.9166 | 0.8101 | 0.5882 |
| *TLR4* | Mm00445273_m1 | <0.0001 | 0.0083 | 0.1835 | <0.0001 |
| *Casp1* | Mm00438023_m1 | <0.0001 | 0.0949 | 0.0018 | <0.0001 |
| *CD14* | Mm00438094_g1 | <0.0001 | 0.0039 | 0.0361 | <0.0001 |
| *TLR2* | Mm00442346_m1 | <0.0001 | 0.0058 | 0.0109 | <0.0001 |
| *Nlrp3* | Mm00840904_m1 | <0.0001 | 0.0537 | 0.0075 | <0.0001 |
| *IL1R2* | Mm00439622_m1 | 0.0076 | 0.4914 | 0.881 | 0.0085 |
| *C3* | Mm01232779_m1 | 0.0003 | 0.0220 | 0.2829 | 0.0004 |
| *C4b* | Mm00437893_g1 | <0.0001 | 0.0066 | 0.0288 | <0.0001 |
| *C3aR1* | Mm02620006_s1 | 0.0029 | 0.1196 | 0.2076 | 0.0042 |
| *C5aR1* | Mm00500292_s1 | 0.0011 | 0.0084 | 0.8032 | 0.0009 |
| *CR1* | Mm00801681_m1 | 0.0822 | 0.3902 | 0.5584 | 0.0994 |
| *Mmp3* | Mm00440295_m1 | 0.0008 | 0.5493 | 0.0032 | 0.0004 |
| *Mmp9* | Mm00442991_m1 | 0.0122 | 0.7313 | 0.0391 | 0.0088 |
| *Tgfb1* | Mm00516117_m1 | <0.0001 | 0.0003 | 0.0565 | <0.0001 |
| *Tgfbr1* | Mm01178820_m1 | <0.0001 | 0.0021 | 0.0033 | <0.0001 |
| *Tgfbr2* | Mm00436971_m1 | <0.0001 | 0.0163 | 0.0018 | <0.0001 |
| *IL1R1* | Mm00436955_m1 | 0.974 | 0.8650 | 0.7019 | 0.7151 |
| *TIMP1* | Mm01341361_m1 | 0.0134 | 0.1687 | 0.4587 | 0.0176 |
| *Col1a1* | Mm00801666_g1 | 0.0415 | 0.2009 | 0.6467 | 0.0506 |
| *Col3a1* | Mm00802300_m1 | 0.1717 | 0.9954 | 0.1118 | 0.0848 |
| *Hprt* | Mm03024075_m1 | n/a | n/a | n/a | n/a |

*Abbreviations*: ANOVA, analysis of variance; *Arg1*, Arginase 1; *C3*, complement component 3; *C4b*, complement component 4b; *C3aR1*, complement component 3 antagonist receptor 1; *C5aR1*, complement component 5 antagonist receptor 1; *Casp1*, Caspase 1; CD, cluster of differentiation; *Chi3l3*, mouse chitinase 3-like 3; *cKit*, tyrosine-protein kinase Kit; *Col1a1*, Collagen Type I Alpha 1; *Col3a1*, Collagen Type 3 Alpha 1; CR, complement receptor; CVB3, coxsackievirus B3 antigen *VP1*; Cxcl, C-X-C motif chemokine ligand; *F4/80*, EGF-like module-containing mucin-like hormone receptor-like 1; *Fizz1*, found in inflammatory zone 1; *FoxP3*, forkhead box P3; *Hprt*, hypoxanthine-guanine phosphoribosyltransferase; *IL1R1*, IL-1 receptor 1; *IL1R2*, IL-1 receptor 2; *Mmp*, matrix metalloproteinase; *Mrc1*, mannose receptor C-Type 1; *Nos2*, nitric oxide synthase 2; *Nlrp3*, NOD- LRR- and pyrin domain-containing protein 3; PEV, platelet-derived extracellular vesicles; pmPEV, pre-menopausal platelet derived extracellular vesicles; *Tgfb1*, tissue growth factor beta 1; *Tgfbr1*, tissue growth factor beta receptor 1; *Tgfbr2*, tissue growth factor beta receptor 2; *TIMP1*, TIMP metallopeptidase inhibitor 1; *TLR*, Toll like receptor.
